# The Role of INAPERTURATE POLLEN1 as a Pollen Aperture Factor Is Conserved in the Basal Eudicot *Eschscholzia californica* (Papaveraceae)

**DOI:** 10.3389/fpls.2021.701286

**Published:** 2021-07-07

**Authors:** Ismael Mazuecos-Aguilera, Ana Teresa Romero-García, Božena Klodová, David Honys, María C. Fernández-Fernández, Samira Ben-Menni Schuler, Anna A. Dobritsa, Víctor N. Suárez-Santiago

**Affiliations:** ^1^Department of Botany, Faculty of Sciences, University of Granada, Granada, Spain; ^2^Laboratory of Pollen Biology, Institute of Experimental Botany of the Czech Academy of Sciences, Prague, Czechia; ^3^Department of Experimental Plant Biology, Faculty of Science, Charles University, Prague, Czechia; ^4^Department of Cell Biology, Faculty of Sciences, University of Granada, Granada, Spain; ^5^Department of Molecular Genetics and Center for Applied Plant Sciences, Ohio State University, Columbus, OH, United States

**Keywords:** *Eschscholzia californica*, INAPERTURATE POLLEN1, Papaveraceae, pollen, pollen aperture, RNA-seq, transcriptome analysis, VIGS

## Abstract

Pollen grains show an enormous variety of aperture systems. What genes are involved in the aperture formation pathway and how conserved this pathway is in angiosperms remains largely unknown. *INAPERTURATE POLLEN1* (*INP1*) encodes a protein of unknown function, essential for aperture formation in Arabidopsis, rice and maize. Yet, because INP1 sequences are quite divergent, it is unclear if their function is conserved across angiosperms. Here, we conducted a functional study of the *INP1* ortholog from the basal eudicot *Eschscholzia californica* (*EcINP1*) using expression analyses, virus-induced gene silencing, pollen germination assay, and transcriptomics. We found that *EcINP1* expression peaks at the tetrad stage of pollen development, consistent with its role in aperture formation, which occurs at that stage, and showed, via gene silencing, that the role of *INP1* as an important aperture factor extends to basal eudicots. Using germination assays, we demonstrated that, in *Eschscholzia*, apertures are dispensable for pollen germination. Our comparative transcriptome analysis of wild-type and silenced plants identified over 900 differentially expressed genes, many of them potential candidates for the aperture pathway. Our study substantiates the importance of *INP1* homologs for aperture formation across angiosperms and opens up new avenues for functional studies of other aperture candidate genes.

## Introduction

Pollen, the male gametophyte of spermatophytes, is surrounded by a robust, sporopollenin-based pollen wall, called exine, which isolates and protects it from the external environment (Ariizumi and Toriyama, 2011). Although most of the pollen surface is covered by exine, in many plants certain regions of the pollen surface receive little to no exine deposition. These regions, known as pollen apertures, represent some of the most characteristic and well-defined elements of the pollen surface (Furness and Rudall, 2004; Zhou and Dobritsa, 2019). Apertures often serve as the sites for pollen tube exit (Heslop-Harrison and Heslop-Harrison, 1985; Edlund et al., 2004; Edlund et al., 2016). They are also involved in the exchange of water and solutes with the medium and allow the rigid exine to adjust to changes in pollen volume due to dehydration/rehydration during pollination (Heslop-Harrison, 1979).

Numerous studies have been carried out to characterize the large diversity of aperture patterns in angiosperms (e.g., Wodehouse, 1935; Blackmore et al., 1995; Furness and Rudall, 2004; Pérez-Gutiérrez et al., 2015; Wortley et al., 2015; Matamoro-Vidal et al., 2016). Although the number, morphology and position of apertures often vary among species, these attributes usually remain stable at the intraspecific level, suggesting a genetic control of aperture patterning which changed multiple times during the evolution of flowering plants. However, few studies so far have probed the molecular mechanisms of aperture formation.

Genetic screening in *Arabidopsis thaliana* has made it possible to identify mutants defective in aperture formation (Dobritsa et al., 2011; Lee et al., 2018; Plourde et al., 2019). One of the genes discovered in these screens, *INAPERTURATE POLLEN1* (*INP1*), encodes a protein of unknown function that is essential for aperture formation (Dobritsa and Coerper, 2012). In the Arabidopsis *inp1* mutant, pollen completely lacks apertures, producing inaperturate phenotype.

During pollen development, Arabidopsis *INP1* (*AtINP1*) first becomes expressed in pollen mother cells, where its protein is evenly distributed in the cytoplasm. Later, at the post-meiotic tetrad stage, INP1 accumulates at the specific plasma-membrane (PM) regions in each microspore (the predecessor of the pollen grain) and assembles into punctate lines at the sites where apertures will be formed (Dobritsa and Coerper, 2012; Dobritsa et al., 2018). Arabidopsis *INP1* reaches peak expression at the tetrad stage, and after the release of microspores from tetrads its expression quickly disappears. At the aperture PM domains, AtINP1 appears to localize at the interface between the PM and the callose wall surrounding the tetrad of microspores, where it may act as a bridge, keeping the aperture domains of each microspore attached to the callose wall to prevent sporopollenin deposition at these regions (Dobritsa et al., 2018). It is not known whether AtINP1 interacts with other proteins to form these bridges (Zhou and Dobritsa, 2019).

Although both aperture patterns and the INP1 protein sequences are quite divergent across angiosperms, the involvement of INP1 proteins in the process of aperture formation seems to be conserved, as indicated by the loss of apertures in the *inp1* mutants of maize and rice (Li et al., 2018; Zhang et al., 2020). Normally, these two monocot species have the ulcerate aperture pattern characteristic of the Poaceae family, with a single pore-like polar aperture, very different from the typical eudicot tricolpate pattern of Arabidopsis pollen, with its three meridional furrows. Also, while the INP1 protein sequences are very similar between maize and rice, they share only ∼35% sequence identity with AtINP1 (Dobritsa and Coerper, 2012; Li et al., 2018). It was, therefore, surprising that these quite divergent proteins are all involved in the same process. Like AtINP1, the rice INP1 (OsINP1) localizes to the aperture PM domains in microspores, assembling into a single ring-like structure that pre-marks the position of a single pore-like aperture (Zhang et al., 2020). Interestingly, although *inp1* pollen in Arabidopsis is fertile, *inp1* pollen in rice and maize loses its ability to germinate pollen tubes and becomes completely sterile, demonstrating that apertures in these species, but not in Arabidopsis, are essential for pollen fertility.

Although the INP1 homologs from several species are all involved in the same process, they cannot readily substitute for each other. The sequence divergence among INP1 homologs was suggested to be responsible for the fact that only proteins from closely related species from Brassicaceae were able to restore apertures in the Arabidopsis *inp1* mutants during the interspecific complementation experiments, while the orthologs from Solanaceae, Papaveraceae, and Poaceae were unable to do it (Dobritsa and Coerper, 2012; Li et al., 2018). Based on these results, the need for species-specific partners to cooperate with INP1 in the control of aperture formation was postulated (Li et al., 2018). However, a functional analysis of the INP1 homologs from more plant lineages is necessary to ascertain that the role of INP1 as an aperture factor is conserved across angiosperms.

In this study, we investigated the functional conservation of the INP1 ortholog EcINP1 from *Eschscholzia californica* (California poppy), a species of Papaveraceae family. This is an early divergent family within the order Ranunculales, the lineage that first diverged from the large group of eudicots (Wang et al., 2009; Hoot et al., 2015). At the base of the eudicot clade there was a transition in the pollen grain aperture system, from pollen with one polar aperture –typical of basal angiosperms and monocots– to pollen with three apertures in equatorial positions, which may have been a key innovation involved in the success and diversification of eudicots (Furness and Rudall, 2004). Because of the phylogenetic position of Ranunculales, between monocots and the core eudicots, and its high pollen aperture system diversity (Blackmore et al., 1995; Pérez-Gutiérrez et al., 2016; Zhang et al., 2017), the Ranunculales species provide the opportunity to study the conservation of genetic mechanisms involved in the aperture system and its evolution. Unlike pollen from the species in which INP1 was previously studied, pollen of *E. californica* develops between five and seven colpate apertures. AtINP1 and EcINP1 share only ∼44% sequence identity, and it was previously shown that EcINP1 cannot substitute for AtINP1 in Arabidopsis (Li et al., 2018). However, it remained unclear if this was due to the EcINP1’s dependence on additional factors from *E. californica* or because EcINP1 is no longer involved in aperture formation. Here, we (1) analyzed patterns of *EcINP1* expression to demonstrate that it is also expressed in anthers during the tetrad stage of pollen development, (2) inactivated *EcINP1* with virus-induced gene silencing (VIGS) to reveal that the *INP1* involvement in aperture formation is conserved in *E. californica*, (3) tested whether pollen germination in this species requires the presence of apertures, and (4) identified genes whose expression was affected by the silencing of *EcINP1*, which might represent candidates involved in the aperture formation mechanisms.

## Materials and Methods

### Plant Material

The seeds of *Eschscholzia californica* Cham. were purchased from the company Seedaholic (Cloghbrack, Clonbur, Galway, Ireland). They were sown in pots (9 × 9 × 9 cm^3^) with universal substrate and vermiculite mixed in a 3:1 ratio and kept in a greenhouse at a temperature range between 26 and 14°C under a light/dark cycle of 16/8 h. Each pot was fertilized once at the beginning of the experiment and watered every day.

### DNA/RNA Extraction, PCR Amplification of *EcINP1* Analysis of Intraspecific Variability, and Phylogenetic Analysis

Genomic DNA (gDNA) was isolated from fresh leaves using the NucleoSpin^®^ Plant II Kit (Macherey-Nagel GmbH and Co., Germany), and total RNA was extracted using the NucleoSpin^®^ RNA Plant kit (Macherey-Nagel GmbH and Co., Germany), following the manufacturer’s instructions. 1 μg of RNA was reverse-transcribed to cDNA using SuperScript III Reverse Transcriptase (Invitrogen, Karlsruhe, Germany) and oligo(dT)18 (ThermoFisher Scientific, United States).

The full-length coding sequence of *EcINP1* was published in Li et al. (2018; ENA accession number LT840341). To study intraspecific variation of *EcINP1*, PCR amplification was carried out on both gDNA and cDNA from bulked individuals (5 individuals for gDNA and 5 for cDNA) using the primers EcaINP1-F and EcaINP1-R (Li et al., 2018; Supplementary Table 1). PCR products were cloned using the Strataclon blunt PCR cloning kit (Agilent Technologies, United States), and 17 cDNA and 10 gDNA clones were sequenced. In addition, we included four sequences from the RNA-seq data generated in our laboratory, the sequence published in Li et al. (2018), one complete sequence from the 1000 Plants Project (1KP; Wickett et al., 2014), and the genomic sequence retrieved from the *Eschscholzia* Genome Database (Hori et al., 2018). The nucleotide sequences were aligned in Bioedit (Hall, 1999) and, after intron removal from the gDNA sequences, the observed genetic distances (*p*-distance) were estimated with MEGA X (Kumar et al., 2018).

For phylogenetic analyses, nucleotide sequences from other Papaveraceae and different angiosperm species were obtained by BLAST searches from NCBI, 1KP, Phytozome^1^, and Phytometasyn (Xiao et al., 2013^2^) databases (Supplementary Table 2). Sequences from the same species with DNA identity of >95% were regarded as possible alleles, so only one sequence was considered. After translating the nucleotide sequences, all the protein sequences were aligned using the Clustal algorithm in Bioedit and adjusted manually. The aligned sequences were used to generate a Maximum Likelihood (ML) tree. The ML analysis was performed with the program PhyML v3.0 (Guindon and Gascuel, 2003) through the web platform “ATCG: Montpellier Bioinformatics Platform,” applying the substitution model JTT automatically selected by Smart Model Selection in PhyML (Lefort et al., 2017) and the Akaike Information Criterion (AIC; Akaike, 1974). The search for the optimal tree was carried out using the subtree-pruning and regrafting algorithm from five random starting trees generated by the parsimony algorithm (Guindon et al., 2010). Branch support was assessed using the approximated likelihood ratio test (aLRT) with the Shimodaira-Hasegawa-like test interpretation (Anisimova and Gascuel, 2006; Guindon et al., 2010).

A ML phylogenetic analysis was also performed to establish the homology relationships of two genes of interest, identified by transcriptome analyses (see below), related to the AGC1 group kinases from Arabidopsis. This analysis was based on the protein sequences of the kinase domain of all AGC1 kinases from Arabidopsis and those sequences obtained by BLAST from the genomes of *E. californica* and *Papaver somniferum*. The sequences of the AGC3-group kinases from Arabidopsis were used as an outgroup. Sequence alignment and ML analysis were conducted as described above.

### Semi-Quantitative Reverse Transcription-Polymerase Chain Reaction (Semi-qRT-PCR) and Quantitative RT-PCR (qRT-PCR)

The spatial pattern of expression of the *EcINP1* gene was tested by semi-qRT-PCR on vegetative organs, floral organs of flowers at anthesis, and immature and mature fruits. The temporal expression pattern in anthers at different stages of pollen development was tested by qRT-PCR. Stages of pollen development were determined by optical microscopy (Olympus-CX31, Japan) using glycerin gelatine with basic fuchsin (50 ml of glycerin, 7 g of gelatine, 1 g of phenol, a few crystals of basic fuchsin, and 42 ml of distilled water) pre-warmed to 30–35°C before staining pollen grains. RNA extraction and cDNA synthesis were performed as described above and cDNA pools were diluted to 50 ng/μl for subsequent semi-qRT-PCR and qRT-PCR analyses. Quantitative RT-PCR was also used to test for reduction in the *EcINP1* expression in the VIGS experiments, to validate the expression levels of eight genes of interest selected with the help of transcriptome analyses, and to analyse the temporal expression pattern of three of these eight genes throughout pollen ontogeny. *ACTIN* served as the reference gene for calculating the relative expression intensities in both semi-qRT-PCR and qRT-PCR analyses, using the 2^Δ^ ^Δ^ ^Ct^ method (Livak and Schmittgen, 2001). Quantitative RT-PCR was performed using the FastGene IC Green 2x qPCR mix (NIPPON Genetics, Tokyo, Japan) according to the manufacturer’s instructions, and the qTower 2.2 real-time PCR thermocycler (Analytik Jena, Germany). Gene-specific primers used for semi-qRT-PCR and qRT-PCR reactions were designed using the software Primer3 (Untergasser et al., 2012; Supplementary Table 1). All experiments were repeated with three biological and three technical replicates.

### Western Blot

Anthers of wild-type plants were collected when pollen grains were at the pre-meiosis, tetrad, microspore and mature stages. For protein extraction, the anthers were crushed in the extraction buffer (50 mM Tris-HCl, 9 M Urea, 1% (v/v) Triton X-100, pH 7.4), and the insoluble material was removed by centrifugation at 10,000 g for 10 min at 4°C. The protein extracts were subjected to sodium dodecyl sulfate–polyacrylamide gel electrophoresis (SDS-PAGE). Protein profiles were determined by means of Stain-free technology using a Gel DocTM EZ System (Bio-Rad; Supplementary Figure 1), and were used as a loading control as described by Welinder and Ekblad (2011) and using AlphaView software (Cell Biosciences, Santa Clara, CA) for protein quantification. Proteins were electroblotted from the gel onto a PVDF membrane in a Semi-Dry Transfer Cell (Bio-Rad), and the membrane was blocked for 1 h at room temperature in Tris-buffered saline (TBS) buffer, pH 7.4 containing 5% (w/v) defatted milk and 0.1% (v/v) Tween-20. Immunodetection of EcINP1 was carried out by incubation with a polyclonal antibody SAN-ESK generated against the epitope ESKQEILKTVEKDLMVEIEE (synthesized by Davids Biotechnologie GmbH, Regensburg, Germany) diluted 1:500 in TBS buffer (pH 7.4) containing 0.3% (v/v) Tween-20, overnight at 4°C. An HRP-conjugated anti-chicken IgY (Abcam, ref. ab97135, Cambridge, United Kingdom), diluted 1:2000, served as the secondary antibody. Protein bands were visualized in a C-Digit scanner (LI-COR Biotechnology, United States).

### Virus Induced Gene-Silencing

The tobacco rattle virus (TRV)-based system (Liu et al., 2002) was used for VIGS experiments. A pTRV2:*EcINP1* construct was made by cloning a 480-bp fragment of the *EcINP1* coding region, amplified with the Phusion High-Fidelity DNA polymerase (ThermoFisher Scientific, United States) and primers EcaINP1-F2-*Eco*RI and EcaINP1-R2-*Bam*HI (Supplementary Table 1), between the *Eco*RI and *Bam*HI restriction sites. pTRV2:*EcINP1* as well as negative- and positive-control constructs (respectively, pTRV2:empty, without any insert, and pTRV2:*PDS*, with a fragment of the phytoene desaturase gene) were transformed into *Agrobacterium tumefaciens* strain pGV3101. Three-week-old plants were inoculated with *Agrobacterium* in the hypocotyl following Tekleyohans et al. (2013) and left at 4°C overnight, after which they were transferred to the greenhouse at a temperature range between 15 and 24°C. 78 plants were infected with pTRV2:*EcINP1*, 15 with pTRV2:*PDS*, and 30 with pTRV2:empty. In addition, 50 non-treated plants were grown to observe the wild-type phenotype.

Pollen phenotypes were observed by optical microscopy in the fully open flowers. For 55 plants, pollen was collected from three flowers and three anthers per flower and stained with glycerin gelatine with basic fuchsin. Phenotypes were classified as follows: wild-type, with normal 5-7-colpate apertures; inaperturate, without apertures; and affected, with abnormal apertures that were shorter or shallower than normal. In addition, anthers from three *EcINP1*-silenced plants and three wild-type plants were fixed according to Fernández et al. (1992) and pollen was observed with a scanning electron microscope (model SMT; Zeiss) at the Centro de Instrumentación Científica (University of Granada). Pollen was also stained with auramine O as previously described (Reeder et al., 2016) and observed with confocal microscopy (Nikon A1+, Japan).

In order to test the effectiveness of VIGS, the expression levels of *EcINP1* were measured by qRT-PCR on the first bud (2–3 mm; pollen at the tetrad/free-microspore stage) of four silenced (one with the affected phenotype and three with the inaperturate phenotype) and three non-silenced (pTRV2:empty; wild-type phenotype) plants, as described above.

### *In vitro* Pollen Germination

Pollen grains were cultured for 2 h on plates with 5 ml of liquid germination medium (15 g sucrose, 30 mg Ca(NO_3_)_2_, 20 mg MgSO_4_, 10 mg KNO_3_, and 10 mg H_3_BO_3_ in 100 ml of distilled water) at 25°C under moist conditions and in the dark. For both wild-type and inaperturate pollen from three wild-type plants and two VIGS plants, we counted 100 pollen grains from three anthers per flower and three flowers per plant. The pollen count was carried out using an inverted optical microscope (ZEISS Axio Scope A1, Germany) and, after checking the normality of the data, an analysis of variance (ANOVA) was carried out using IBM SPSS statistics (SPSS Inc., IBM Company, 2020).

### Transcriptome Sequencing, RNA-seq Data Analysis and Annotation

RNA from anthers with pollen at the free-microspore stage was extracted from three inaperturate and three wild-type plants as described above. The extracts were sent to Macrogen Inc. (Korea), where libraries were prepared using the Illumina TruSeq Stranded Total RNA Sample Preparation Kit with Ribo-Zero Plant and sequenced on an Illumina Hiseq 2500 platform as 150 nucleotides paired-ends. Raw data was generated using the Illumina package bcl2fastq.

Raw single reads (in FASTQ format) were subjected to sequence quality control using FastQC v0.11.8^3^. Adaptors and low-quality sequences were removed from the data set using TrimGalore v0.6.4^4^ and a minimum quality score of 20. The library’s characteristics and trimming efficiency were checked and reads aligned to the genome of *Eschscholzia californica*^5^ using the software HISAT2 v2.1.0^6^ (Pertea et al., 2016). Transcripts were assembled and quantified using the software StringTie v2.0^7^, with the merge option (Pertea et al., 2016). Analysis of differentially expressed genes (DEGs) was performed with the DESeq2 R package (v1.24.0) using the coverage produced by StringTie. DEGs were annotated by BLASTX search against the genome of *Papaver somniferum*^8^ and the SwissProt Database^9^, using default parameters and extracting only the top hit for each sequence. To assign a function to each DEG, annotated DEGs were further annotated with Gene Ontology (GO, Ashburner et al., 2000) and Kyoto Encyclopedia of Genes and Genomes (KEGG, Kanehisa and Goto, 2000) databases using the Blast2GO v5.2.5 application (Götz et al., 2008) and the GhostKoala mapping tool (Kanehisa et al., 2016), respectively. To predict putative plant transcription factors (TF), coding sequences were aligned to TF domains from the Plant Transcription Factor Database (PlantTFDB, Zhang et al., 2011).

To validate the gene expression results, qRT-PCR experiments were performed for eight DEGs. These genes were selected because their predicted functions make them interesting candidates for a role in pollen aperture formation. The primer sequences used are listed in Supplementary Table 1.

### Data Availability

All raw sequences for transcriptome are available in the European Nucleotide Archive (ENA) under the accession numbers ERS6376182-ERS6376184 for wild-type samples and ERS6376185-ERS6376187 for VIGS-treated samples.

## Results

### Intraspecific Variability and Phylogeny of *EcINP1*

INP1 is a novel protein whose structure and domain organization are not well understood. Previously, AtINP1 was divided into five regions, based on their evolutionary conservation and position relative to the single predicted domain in this protein, the domain of unknown function DOG1 (Li et al., 2018). To better understand the relative importance of different INP1 regions, we explored the extent of the intraspecific variation of the EcINP1 sequences. To this end, we amplified and analyzed the gDNA and cDNA sequences from multiple plants of *E. californica*, as well as retrieved information from sequence databases. In total, we obtained 34 different *EcINP1* sequences: 31 by us (17 from cDNA, 10 from gDNA, and four from our RNA-seq data) and three from databases (two transcriptomic and one genomic sequences). After *in silico* translating these sequences, we found that five cDNA sequences had substitutions resulting in stop codons, and those were removed from the alignment and not analyzed further. The combination of sequences obtained from cDNA and gDNA made it possible to determine the location of an intron near the beginning of the coding sequence (as in *INP1* orthologs from several other species), as well as to discover a possible second intron, which was found in four cases near the very end of the coding sequence, suggesting the existence of a second possible transcript isoform (Supplementary Figure 2).

The intraspecific variation of *EcINP1* was low. The mean observed genetic distance between sequences was 0.92%, with a total of 34 variable nucleotide positions. In 11 sequences, an insertion of one triplet (AAT) was present at the beginning of the acidic domain (one of the protein regions proposed for AtINP1 by Li et al., 2018). All pairwise comparisons were always at more than 97.5% sequence identity. At the protein level, the number of variable positions was 17, distributed within the protein regions as follows: 3 N-terminal; 5 DOG1; 3 acidic; 2 middle; 4 C-terminal. The sequences with the extra triplet in the acidic region contained an extra asparagine, and one of the transcripts included three additional amino acidic residues at the end of the C-terminal domain (Supplementary Figure 2, sequence 5).

Curiously, in our intraspecific library of *EcINP1* we uncovered a sequence with significant homology to multiple dispersed *EcINP1* fragments that also included 255 bp of the ubiquitin-like domain-containing CTD phosphatase (Supplementary Figure 3, *EcUBCL1*). Portions of this sequence correspond to fragments from the *5’UTR*, the N-terminal region, the DOG1 domain, the acidic region, as well as portions of the C-terminal region and the 3’UTR of *EcINP1* (Supplementary Figure 3). This sequence was also found by BLAST searches in the GenBank (JG611242), the 1KP (TUHA-2032598, NJKC-2028667, RKGT-2014503, EVOD-2107709), and the *Eschscholzia* Genome (Eca_sc001433.1_4218210.424882) databases. We performed a VIGS experiment to silence this sequence, using a 493 bp region that included the ubiquitin-like domain-containing CTD phosphatase region (Supplementary Figure 3; see Supplementary Table 1 for primer sequences), but did not observe any abnormal phenotypes in the treated plants (data not shown), so the functional significance of this chimeric sequence remains unknown.

**FIGURE 1:**
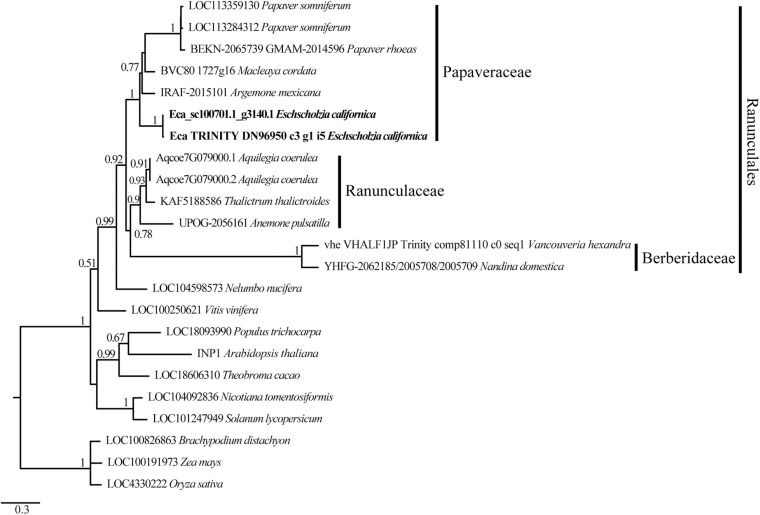
Maximum likelihood phylogenetic tree of INP1-like protein sequences showing EcINP1 homology relationships of *Eschscholzia californica*. The name of each sequence is composed of the locus identifier followed by the species name. Sequence accession numbers can be found in Supplementary Table 2. The EcINP1 sequences representing two protein isoforms are in bold type. The families to which the selected sequences from the order Ranunculales belong are indicated on the right.

To verify the relationships of the *EcINP1* gene with *INP1* homologs from other plants, we conducted a phylogenetic analysis using sequences from other members of Papaveraceae and from other angiosperm species. The ML tree shows that EcINP1 is grouped with the rest of INP1 sequences from Papaveraceae, which all fall into the Ranunculales clade, together with sequences from Ranunculaceae and Berberidaceae (Figure 1). In general, the INP1 tree follows a pattern according to the angiosperm phylogeny.

### *EcINP1* Is Expressed in Anthers During Pollen Aperture Development

**FIGURE 2:**
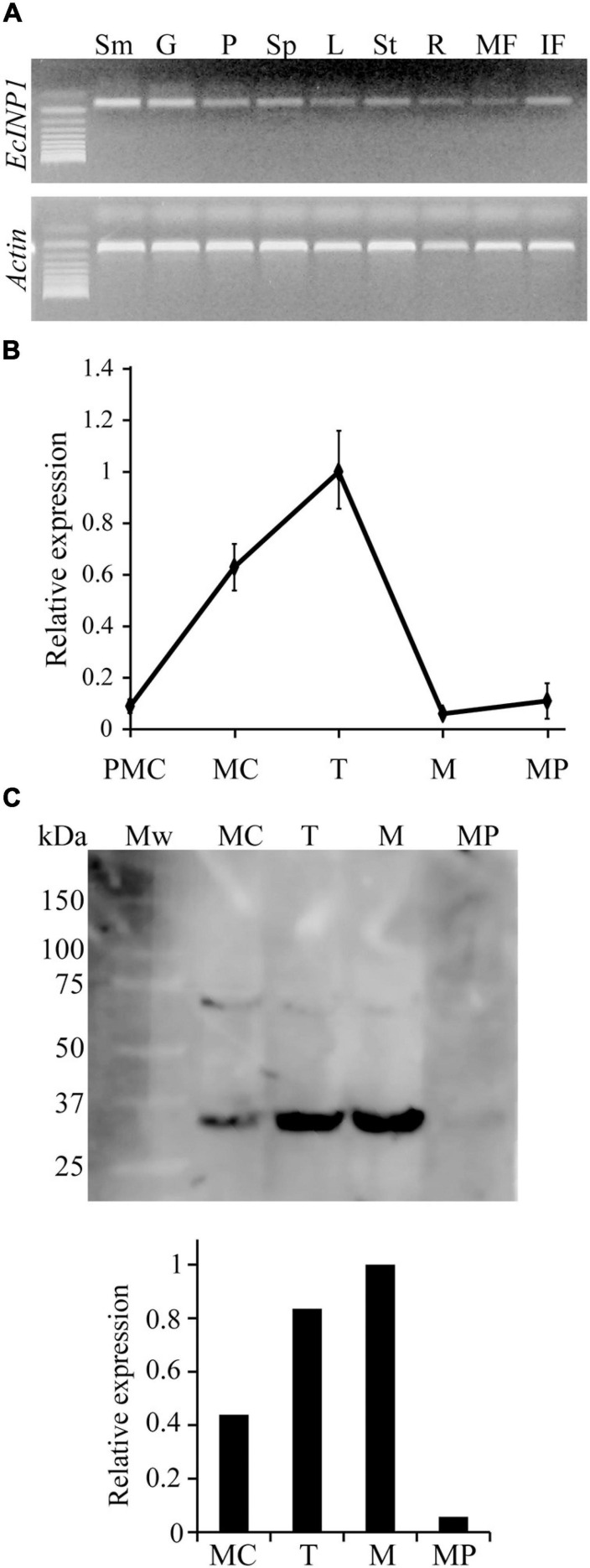
Expression patterns of *EcINP1*. **(A)** Expression analysis of *EcINP1*, based on semi-quantitative RT-PCR, in different plant organs. Sm, stamen; G, gynoecium; P, petal; Sp, sepal; L, leaf; St, stem; R, root; MF, mature fruit; IF, immature fruit. **(B)** qRT-PCR-based expression analysis of *EcINP1* in anthers at different pollen development stages. Actin was used as a normalization control. **(C)** Detection of EcINP1 protein levels by Western blot immunoassay in anthers at different pollen development stages. The bar plot shows the relative expression of EcINP1 (normalized to total proteins detected with a Stain-free technology gel as a loading control and quantified with the AlphaView software). PMC, pre-mother cell; MC, mother cell; T, tetrad; M, microspore; MP, mature pollen.

To test where EcINP1 is expressed, we performed a semi-qRT-PCR on multiple plant organs. *EcINP1* transcripts were found in all tested vegetative, floral, and fruit organs. The highest signal intensity was observed in stamens, followed by the gynoecium (Figure 2A).

We then used qRT-PCR to assess the pattern of temporal expression of *EcINP1* in anthers during different stages of pollen ontogeny (Figure 2B). The *EcINP1* expression starts at the end of the microsporogenesis process, during the pre-mother cell stage. It reaches maximal levels during the tetrad stage, and essentially disappears during the microspore stage. We have also developed an antibody against an epitope in the middle region of EcINP1. Western blot analysis on protein extracts from anthers at different stages revealed a main band with a molecular weight corresponding to the ∼35 kDa expected for EcINP1, and a secondary band that might represent protein dimers. This analysis showed that EcINP1 protein was highly expressed at the tetrad stage and was still present at high concentration at the free-microspore stage (Figure 2C), suggesting that the protein may persist longer than the transcript. Taken together, these results indicate that EcINP1 is produced in developing pollen at the stages concurrent to aperture development.

### *EcINP1* Is Required for the Formation of Pollen Apertures

To test whether the INP1 involvement in aperture formation is conserved in *E. californica*, we attempted to silence *EcINP1* using the tobacco rattle virus (TRV)-based VIGS system (Liu et al., 2002). To this end, we infected plants with the pTRV2:*EcINP1* construct containing a 480-bp fragment of the *EcINP1* coding region.

The silencing of *EcINP1* did not affect vegetative growth, branching, or leaf morphology in any of the 78 plants treated with pTRV2:*EcINP1*, compared to controls (untreated plants and plants treated with the empty vector). 55 of the 78 pTRV2:*EcINP1* plants flowered and did not show any variation in the floral-organ phenotypes.

**FIGURE 3:**
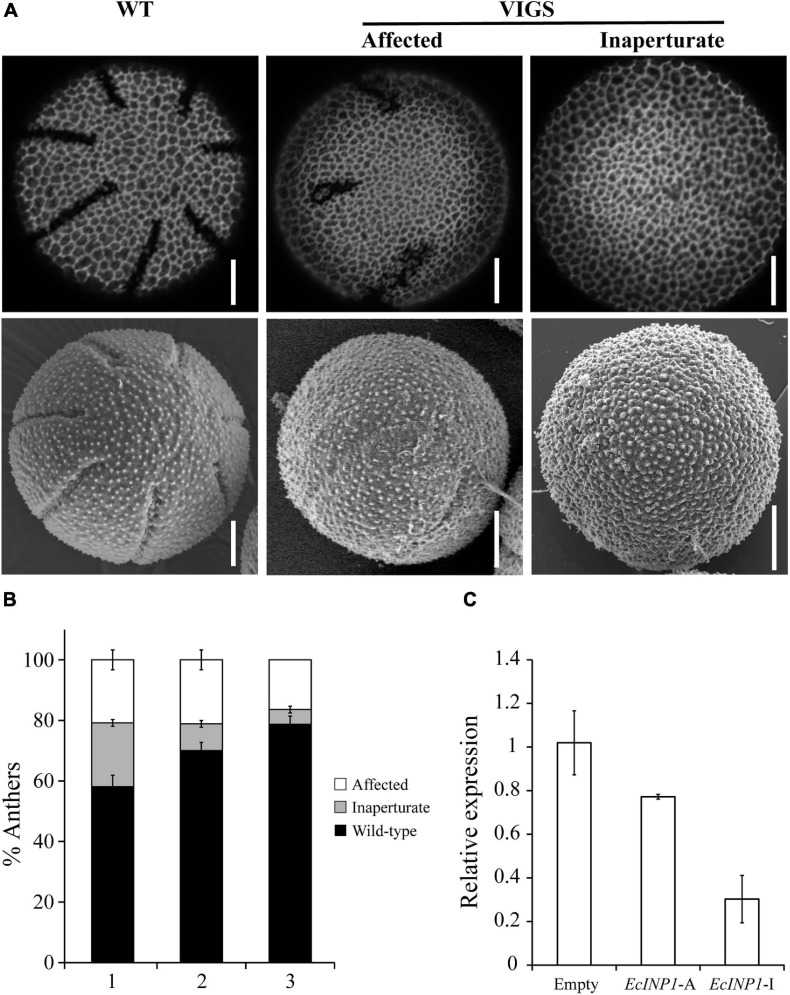
Results of *EcINP1* silencing by VIGS. **(A)** Images showing the observed pollen phenotypes: normal aperturate wild-type (WT), on the left; affected, with shorter and/or shallower apertures, in the middle; inaperturate, on the right. Top images were taken with a confocal microscope and bottom images with a scanning electron microscope. Scale bars = 0.5 μm **(B)** Stacked bar plot showing the distribution of observed phenotypes (black: wild-type, gray: inaperturate, white: affected) according to the developmental order of the three flowers (second = (1), third = (2), and fourth = (3)) sampled for each plant treated with pTRV2:*EcINP1*. Quantification of phenotypes is shown as the percentage of anthers with pollen of each phenotype, since within each anther all pollen grains showed the same phenotype. **(C)** Comparison of *EcINP1* expression, by qRT-PCR analysis of the first flower bud, in plants treated with pTRV2:empty (*n* = 3) and pTRV2:*EcINP1*, differentiating between the affected (*EcINP1*-A*; n* = 1) and inaperturate (*EcINP1*-I*; n* = 3) phenotypes. ANOVA test *P*-value = 0.015.

Yet, when we examined their pollen, we found that their aperture phenotypes showed significant abnormalities. We observed pollen from 139 flowers (34 plants produced three flowers, 16 plants – two flowers, and 5 plants – one flower), collecting three anthers per flower, so, in total, pollen from 417 anthers was analyzed. Within each anther, all pollen presented the same phenotype, but, occasionally, the phenotypes differed between pollen grains from different anthers of the same flower. 33.3% of the observed anthers in pTRV2:*EcINP1* plants presented pollen with some aperture defect (Table 1), compared to 0% in wild-type plants or in those infected with the empty vector. In pTRV2:*EcINP1* plants, 15.3% of anthers had pollen that completely lacked apertures (inaperturate phenotype), while 18% of anthers showed pollen with apertures that were shorter or shallower than normal (affected phenotype) (Table 1; Figure 3A). This result provides strong evidence that, like its homologs from Arabidopsis, rice and maize, EcINP1 is involved in the formation of pollen apertures.

**TABLE 1:** Summary of VIGS experiments to silence the *EcINP1* gene of *Eschscholzia californica*.

	**pTRV2:*EcINP1***	**pTRV2:empty**	**Wild-type**
No. of plants observed	78	30	50
No. of flowering plants	55	24	36
No. of anthers observed	417	180	246
No. of anthers with wild-type pollen	278 (66.6%)	180 (100%)	246 (100%)
No. of anthers with inaperturate pollen	64 (15.3%)	0	0
No. of anthers with affected pollen	75 (18%)	0	0

Since we tracked the developmental order of the flowers collected from each plant, we noticed that the occurrence of anthers with inaperturate pollen often correlated with the order of flower development, decreasing from the oldest flower (the second in development, where the first bud was used to extract RNA) to the youngest flower (the fourth) in the same silenced plant (Figure 3B). This was likely due to the decrease of the silencing effect as flowering progressed (Wege et al., 2007).

qRT-PCR performed on young buds confirmed the downregulation of *EcINP1* in the pTRV2:*EcINP1* plants, with an approximately two-fold reduction in the *EcINP1* levels in these plants compared to the plants infected with the empty vector (Figure 3C).

### Apertures in *E. californica* Are Dispensable for Pollen Germination

**FIGURE 4:**
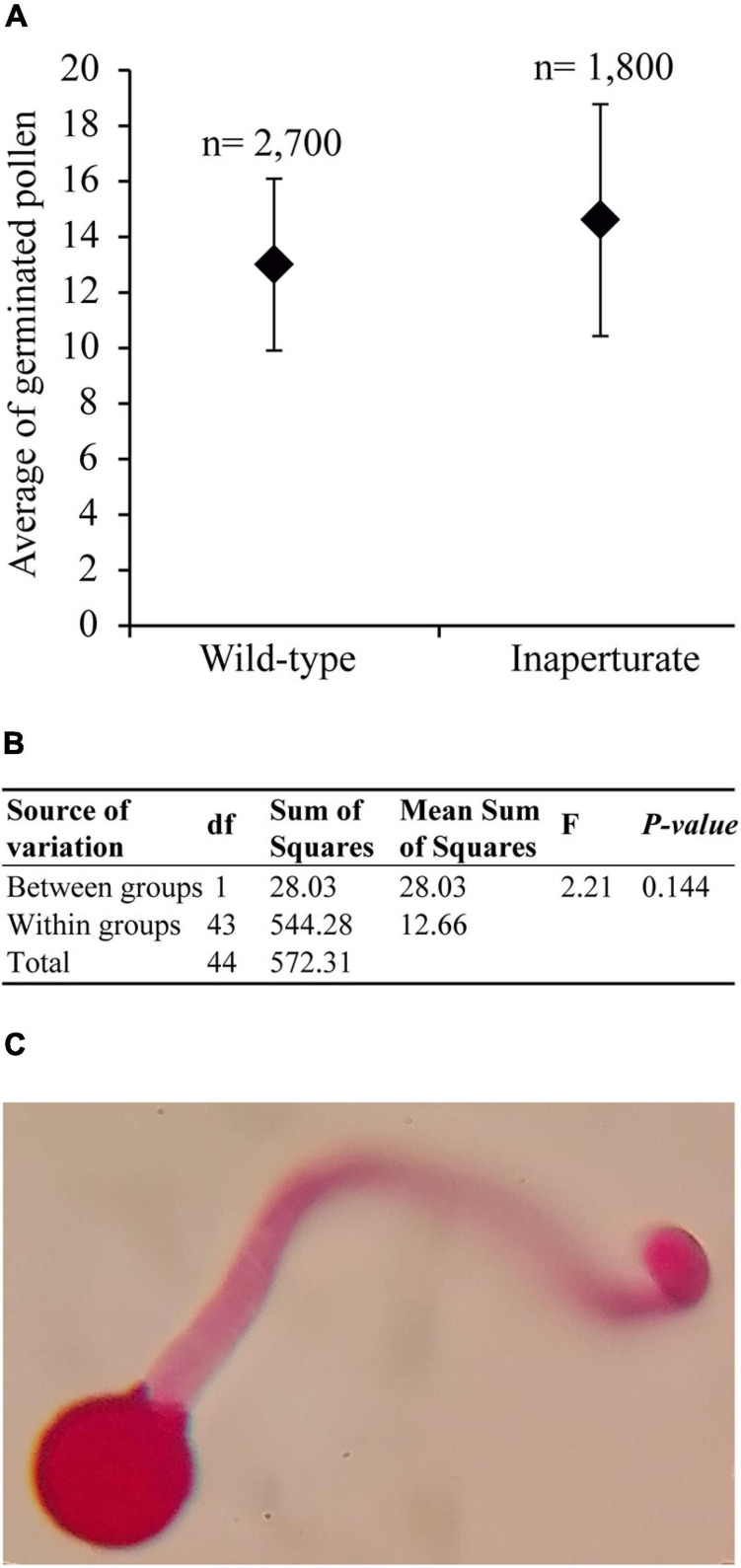
Results of the *Eschscholzia californica* pollen germination test. **(A)** Scatter plot of the mean number of germinated pollen grains per each 100 pollen grains counted, for wild-type and inaperturate pollen. n, total number of pollen grains counted. **(B)** Summary table of the ANOVA analysis. **(C)** Optic microscope image of a germinated inaperturate pollen, stained with basic fuchsin, with the pollen tube emerging through the pollen wall.

As mentioned earlier, pollen apertures are usually thought to serve as the sites for pollen tube exit during germination. Consistent with this notion, inaperturate pollen grains in rice and maize lose their ability to germinate (Li et al., 2018; Zhang et al., 2020). However, in Arabidopsis, *inp1* pollen still shows normal fertility in the absence of apertures (Dobritsa et al., 2011; Albert et al., 2018). Therefore, using an *in vitro* germination test, we tested whether apertures were essential or dispensable for pollen tube germination in *E. californica*. No significant difference was found between the germination of wild-type pollen and the inaperturate pollen from the silenced plants (Figures 4A,B). Thus, the presence of apertures is not essential for pollen germination, and pollen tubes in *E. californica*, like those in Arabidopsis, are capable of emerging directly through the pollen wall (Figure 4C).

### Transcriptome in the *EcINP1*-Silenced Plants Changes Significantly

To determine if silencing of *EcINP1* could affect expression of any genes, we performed RNA-seq analysis of the anthers from the pTRV2:*EcINP1* VIGS plants and compared them with the results for the wild-type plants. Sequencing, using the Illumina platform with Phred quality score, yielded an average of 50,566,911 high-quality (HQ) reads per sample (Supplementary Table 3), after initial quality filtering. On average, 53.53% of reads were aligned to the *E. californica* reference genome and assembled into 34,729 contigs with an average length of 1,006 bp (Supplementary Table 3).

**FIGURE 5:**
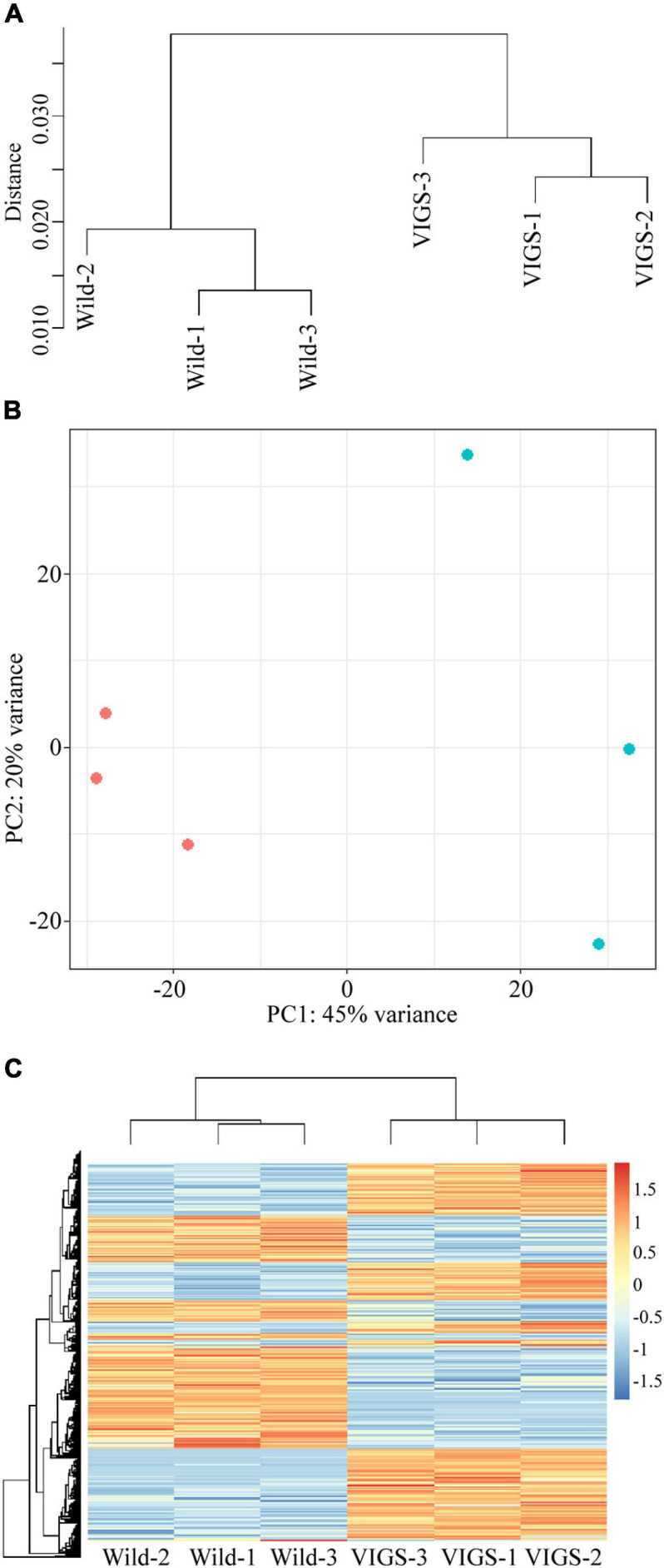
Analysis of differentially expressed genes (DEGs) between transcriptomes of wild-type and VIGS-treated plants. **(A)** Hierarchical clustering shows dissimilarity among the transcriptome samples; distance is calculated by Pearson correlation coefficient. **(B)** Principal component analysis of the transcriptome samples. Red points represent wild-type plants; blue points represent pTRV2:*EcINP1*-treated plants. **(C)** Heatmap of transcriptomes of wild-type and pTRV2:*EcINP1*-treated plants. Heatmap scale bars indicate log_2_fold changes.

**FIGURE 6:**
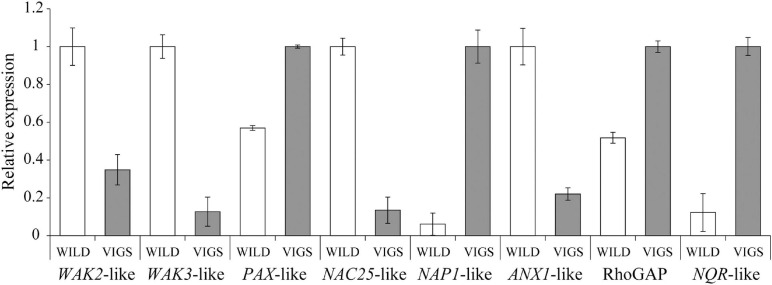
Results of the qRT-PCR analysis to confirm the differential expression of eight DEGs detected through the transcriptome analysis in *Eschscholzia californica*. *WAK2*-like, Eca_sc194563.1_g0010.1; *WAK3*-like, Eca_sc000051.1_g1410.1; *PAX*-like, Eca_sc095603.1_g0010.1; *NAC25*-like, Eca_sc194734.1_g0710.1; *NAP1*-like, Eca_sc004324.1_g2710.1; *ANX1*-like, Eca_sc194691.1_g0370.1; Rho GTPase-activating protein (RhoGAP), Eca_sc009796.1_g0090.1; *NADPH:quinone oxidoreductase* (*NQR*)-like, Eca_sc194522.1_g0180.1.

The hierarchical clustering, heatmap and principal component analyses showed that there were significant differences between the wild-type and VIGS samples (Figures 5A–C). The filtering of the genes with the log_2_-fold change of > 2 and with the *p*-value and *p*-adj of <0.05 identified 971 differentially expressed genes (DEGs), of which 488 were upregulated and 483 were downregulated. As expected, *EcINP1* itself was one of the DEGs, showing downregulation in the VIGS samples with a log_2_-fold change of 2.49, consistent with the result of the qRT-PCR. Out of the 971 DEGs, 211 were annotated through BLASTX with the genome of *Papaver somniferum*, 677 with SwissProt Database, and the remaining 83 did not give hits in any database (Supplementary Table 4). As transcription factors (TFs) play important roles in plant development, we used the Plant Transcription Factor Database (PlantTFDB) to align DEGs with TF domains. 30 DEGs showed high homology to 16 known TF families, among which the NAC family, with 8 genes, was the most represented (Supplementary Table 4 and Supplementary Figure 4A). With respect to functional characterization, 277 (28.5%) of 971 DEGs were classified using GhostKoala into different functional categories, with proteins involved in processing of genetic information being the most common category, followed by proteins involved in carbohydrate metabolism and in signaling and cellular processes, all processes that could be relevant to aperture formation (Supplementary Figure 4B and Supplementary Table 4). We further describe the identity of some of the most interesting DEGs in the Discussion. For eight of those genes, their differential expression was confirmed with qRT-PCR (Figure 6).

### Phylogenetic and Expression Analyses of Three Interesting DEGs

Among the DEGs, we found two upregulated transcripts of the AGCVIII protein serine-threonine kinases from the AGC1 group (in the nomenclature of Galván-Ampudia and Offringa, 2007), putatively annotated as D6 PROTEIN KINASE-LIKE1 (D6PKL1; scaffold Eca_sc004639.1_g0030.1) and D6 PROTEIN KINASE-LIKE2 (D6PKL2; Eca_sc095603.1_g0010.1). In Arabidopsis, the protein D6 PROTEIN KINASE-LIKE3 (D6PKL3) belonging to the same group of kinases is involved in aperture formation (Lee et al., 2018). To clarify the homology relationships of the two D6PKL genes found in our DEG set, we carried out phylogenetic analysis on sequences of the AGC1 group kinases from Arabidopsis, *E. californica* and *Papaver somniferum*. The DEG sequence Eca_sc095603.1_g0010.1 was not included in the analysis due to its incompleteness in the database. However, it appears to be identical to Eca_sc000153.1_g1520.1, which was used instead.

This analysis showed two distinct clades within the D6PK family (Supplementary Figure 5), suggesting a duplication event and a posterior divergence before the eudicot diversification from their common ancestor, with one strongly supported clade, including the Arabidopsis D6PK, D6PKL1, D6PKL2 and their orthologs, and a more weakly supported clade, which included D6PKL3 and the rest of the *E. californica* and *P. somniferum* sequences. Eca_sc004639.1_g0030.1, affected by the *EcINP1* silencing, belongs to this second clade, falling into a subclade unique to the Papaveraceae. The Papaveraceae sequences in the first clade are clearly related to D6PKL2, suggesting that an ancestral D6PKL2 sequence diverged from the lineage leading to D6PK-D6PKL1 in the ancestor of the eudicots. In Arabidopsis, D6PKL3 is the most diverged sequence in the D6PK family. In *E. californica*, the DEG sequence Eca_sc004639.1_g0030.1 is also clearly differentiated from the other members of the family (Supplementary Figure 5). The second D6PK family related DEG, Eca_sc000153.1_g1520.1, is clearly linked to a close relative of the D6PK family, the Arabidopsis gene At2g44830, encoding PROTEIN KINASE ASSOCIATED WITH BRX (PAX) (Supplementary Figure 5).

Another DEG we considered interesting was the homolog of *NUCLEOSOME ASSEMBLY PROTEIN 1* (*NAP1*). In dioecious wild grapevine *Vitis vinifera* subsp. *sylvestris*, this gene, together with the *INP1* homolog, is part of the sexual locus whose members are functional/present in the male but not the female genome (Badouin et al., 2020). To find out if NAP1 and the two AGC1 kinases (D6PK-like and PAX-like) could be acting simultaneously with INP1, we analyzed their transcript expression at different stages of pollen development. The results showed that all three genes reach maximal expression levels during the tetrad stage (Supplementary Figure 6), mimicking the expression pattern of *EcINP1* (Figure 2B).

## Discussion

The molecular mechanisms involved in the formation of pollen apertures remain largely unknown. Studies in this area have so far been mostly carried out in two model species, the core eudicot *A. thaliana* (Brassicaceae, with tricolpate pollen) and the monocot *O. sativa* (Poaceae, with ulcerate pollen). This study is the first functional characterization of an *INP1*-like gene in a basal eudicot, *E. californica* (Papaveraceae, Ranunculales, with penta- to heptacolpate pollen), and therefore, in the evolutionary context, it complements our understanding of the role of this gene in the diversification of the angiosperm apertural system. Moreover, this is the first study in which a comparative transcriptome analysis was performed for identifying potential candidate genes involved in aperture formation, calling for further functional studies.

Our functional study showed that *EcINP1* is required for the formation of apertures in *E. californica*, similar to its homologs in Arabidopsis, maize and rice. This result extends the conserved role of the gene to basal eudicots and supports the hypothesis that INP1 was involved in the aperture formation in the monocot-eudicot common ancestor (Li et al., 2018). In the interspecies complementation experiments conducted by Li et al. (2018), EcINP1 failed to restore apertures in the Arabidopsis *inp1* mutants. Our results now demonstrate the functionality of EcINP1 and support sequence divergence as the cause of this failure.

At the intraspecific level, we have detected a single copy of *EcINP1* in *E. californica*, as deduced from BLAST searches in the *Eschscholzia* genome database and from the high identity among the observed variants of the gene (>97.5%). One of the most variable regions of the gene and protein is the very end of the C-terminus, where we even detected a second intron and a shift in the reading frame resulting in extra amino acids (Supplementary Figure 2). Li et al. (2018) showed that this region was dispensable for the formation of the punctate INP1 lines and apertures in Arabidopsis, and that it was poorly conserved among the INP1 homologs from other species. This evidence suggests that the low functional significance of the C-terminal region leads to relaxation of the selective pressure acting on it, allowing its divergence at the sequence level even within a species. At the interspecific level, this region is highly variable and had to be excluded from the phylogenetic analysis due to alignment ambiguity. In addition, a 30-bp region located in the acidic region was also excluded because of its high variability. This region, together with the DOG1 domain and the middle region, is part of the INP1 central region, essential for the function and stability of the protein and containing amino acids critical for species-specific interactions (Li et al., 2018). However, whether the acidic region itself is involved in species-specific interactions is not known. At the intraspecific level, we have detected moderate variation in the acidic region, with three amino acid substitutions and an indel of an asparagine. We have also detected intraspecific changes in other important areas of the central portion of EcINP1 (five in the DOG1 domain and two in the middle region). Further studies will be required to understand whether these changes in the regions critical for species-specific interactions could be related to the observed variability of the pollen aperture system in *E. californica* (e.g., in the number of apertures or their length).

The gene expression pattern of *INP1* homologs during microsporogenesis also seems to be conserved across angiosperms. Similar to its counterparts in Arabidopsis and rice (Dobritsa and Coerper, 2012; Zhang et al., 2020), *EcINP1* begins expression in pollen mother cells, reaches the maximum level at the early tetrad stage, and, after the release of microspores, its transcript practically disappears. In Arabidopsis, the AtINP1 protein signal disappears soon after the release of microspores from the tetrad, suggesting rapid degradation of the protein (Dobritsa and Coerper, 2012; Dobritsa et al., 2018). The EcINP1 protein, unlike AtINP1, reaches its maximum concentration during the free microspore period (Figure 2C), so degradation of the protein does not occur until after that stage. A delay in the degradation of OsINP1 has also been documented in rice and could be associated with the functional diversification of that protein, which, together with the lectin receptor-like kinase (RLK) DEFECTIVE IN APERTURE FORMATION1 (OsDAF1) with which it interacts, is involved in the formation of the pore annulus (Zhang et al., 2020). Thus, a delay in the EcINP1 degradation beyond aperture formation may suggest a possibility of its functional diversification.

Our pollen germination assay showed that apertures in *E. californica*, like those in Arabidopsis, are not essential for the exit of pollen tubes, and that pollen tubes can break through exine in inaperturate pollen grains. This is different from grasses, in which inaperturate mutants fail to germinate pollen tubes and show complete male sterility (Li et al., 2018; Zhang et al., 2020). Differences in exine morphology (thickness and tectum sculpture; Li et al., 2018) as well as in physiology of pollen and stigma (Edlund et al., 2016) have been proposed as possible causes for differences in dependence of pollen tubes on the presence of apertures. Inaperturate sterile pollen in dioecious species, produced by female flowers and acting as a reward or attractant for pollinators, is a character that has independently evolved at least six times among eudicots (Furness, 2007). Recently, Badouin et al. (2020) found that in female flowers of *Vitis vinifera* subsp. *sylvestris* the *INP1* homolog has an 8-bp deletion in the DOG1 domain, which results in a premature stop codon and is probably responsible for the absence of apertures. However, they did not study whether the lack of apertures in this pollen is sufficient to cause sterility. In this taxon, *INP1* is located within the sex locus, where four other genes, present in male plants, are missing in the female plants and could also be the candidates for pollen sterility. Interestingly, we discovered one of these four genes, *NUCLEOSOME ASSEMBLY PROTEIN 1* (*NAP1*), as part of the DEG set in the *EcINP1*-silenced plants, suggesting that a possibility of its interaction with *INP1* should be examined. Consistent with this notion, we found that *EcNAP1*-like is most strongly expressed during the tetrad stage of pollen ontogeny (Supplementary Figure 6A), when *EcINP1* expression is also maximal and when the process of aperture development begins.

INAPERTURATE POLLEN1 (INP1) is an essential factor for the development of apertures, but it is not the main factor defining aperture number, positions, and morphology (Reeder et al., 2016; Dobritsa et al., 2018; Zhou and Dobritsa, 2019). Still, in Arabidopsis, it has been observed that there is a relationship between the INP1 levels and aperture length, with the lower transcript levels correlating with shorter apertures (Dobritsa and Coerper, 2012). In *E. californica*, this relationship also appears to exist, since older VIGS-silenced plants, in which *EcINP1* was likely inactivated only partially, often produced pollen with apertures that were shorter or shallower than normal (Figure 3B). However, in no case were the changes in number, position or shape of apertures observed.

Although accumulating evidence indicates the need for species-specific partners to cooperate with INP1 in different species to control the formation of apertures (Li et al., 2018; Zhou and Dobritsa, 2019; Zhang et al., 2020), these other molecular players remain largely unknown. Our transcriptome analysis presents a basis for identifying DEGs that may represent some candidates with which EcINP1 interacts. Among the 971 DEGs, we found two belonging to the AGC1 kinases: one related to D6PKL3 of Arabidopsis and the other to PAX. In Arabidopsis, the four membrane-associated kinases of the D6PK family directly regulate the PIN-FORMED (PIN)-mediated auxin transport required for phototropic responses (Zourelidou et al., 2009). However, D6PKL3 is also involved in pollen aperture formation (Lee et al., 2018). D6PKL3 appears to act upstream of INP1, possibly specifying domains in the PM to indicate the sites where INP1 must attach. At the same time, INP1 also seems to control D6PKL3 localization at the aperture domains (Lee et al., 2018). Similar to the D6PK-family proteins, PAX regulates the activity of PIN1 in developing protophloem sieve elements (Marhava et al., 2018). Marhava et al. (2020) have shown that PAX and its partner BREVIS RADIX (BRX) influence the local abundance of PIN1 by recruiting phosphatidylinositol-4-phosphate 5-kinases (PIP5Ks) to partition the PM into distinct domains. In the Arabidopsis aperture formation process a possible link between AGC1 kinases and phosphatidylinositol lipids in the aperture PM domains was also proposed (Lee et al., 2018). The upregulation of the transcripts of these AGC1 kinases in the *E. californica* VIGS-silenced plants suggests their direct or indirect interaction with EcINP1. The temporal expression pattern of these kinases, coincident with that of *EcINP1* (Supplementary Figures 6B,C; Figure 2B), provides further evidence pointing toward the connection between these genes in determining the formation of apertures. Thus, further studies will be necessary to test possible roles of D6PK-like and PAX-like in the establishment of the PM domains of future apertures and EcINP1 polarization.

Among the annotated DEGs, we also found several RLKs. In rice, the above-mentioned lectin RLK OsDAF1 was recently shown to be involved in the formation of pollen apertures (Zhang et al., 2020). Interestingly, one of the upregulated DEGs was annotated as a G-type lectin S-receptor-like serine/threonine-protein kinase (homologous to the Arabidopsis At2g19130). Also, among the downregulated genes we found homologs of two *WALL-ASSOCIATED RECEPTOR KINASES* (*WAK*), *WAK2*, and *WAK3* (Figure 6), encoding cell wall-associated RLKs (He et al., 1999). In *Oryza sativa*, a WAK-RLK gene *DEFECT IN EARLY EMBRYO SAC1* (*OsDEES1*) was shown to have some effect on pollen viability and pollen tube growth (Kohorn, 2001; Wang et al., 2012).

In addition, ten other RLKs were found in our DEG set. Among them was a downregulated homolog of the pollen-specific RLK *ANXUR1* (*ANX1*) (Figure 6). In Arabidopsis, ANX1, together with its homolog ANX2, controls cell wall integrity and pollen tube rupture by regulating pollen-expressed NADPH oxidases, as well as exocytosis and secretion of cell wall materials (Miyazaki et al., 2009; Boisson-Dernier et al., 2009; Fehér and Lajkó, 2015). Related to the possible role of ANX1 in aperture formation, we also found an upregulated homolog of *NADPH:quinone oxidoreductase* (*NQR*) (Figure 6), proposed to be a part of the ANX pathway (Fehér and Lajkó, 2015). Also, we found an upregulated DEG coding for the homolog of the leucine-rich repeat RLK PXY-LIKE1 (PXL1), very closely related to PXY which helps to maintain cell polarity required for the orientation of cell division during vascular development (Fisher and Turner, 2007). Since cell polarity in developing microspores likely plays a role in the establishment of aperture domains (Lee et al., 2018; Zhou and Dobritsa, 2019), polarity-related proteins could be good candidates for functional testing in the future. Additionally, among the upregulated DEGs, we found an uncharacterized Rho GTPase-activating protein (RhoGAP) (Figure 6). Rho GTPases, acting as molecular switches that cycle between the inactive cytosolic GDP-bound state and the active membrane-bound GTP-state, have been implicated in the control of cell polarity, cellular domain formation, and cytoskeletal organization (Craddock et al., 2012), all of which could be important for the formation of aperture domains.

So far, nothing is known about the factors regulating expression of genes involved in pollen aperture formation. Among the annotated DEGs, we found 30 that correspond to transcription factors. Eight belong to the NAC family, whose members regulate many developmental processes in plants. One of them, downregulated in the VIGS plants (Figure 6), is annotated as a homolog of *NAC TRANSCRIPTION FACTOR 25* or *TAPNAC*, known to be expressed in the tapetum of the Arabidopsis anthers (Alvarado et al., 2011).

In summary, our study extends INP1 involvement in aperture formation to basal eudicots. This functional conservation is quite remarkable, given the low protein conservation of INP1 and the large variations in aperture patterns across angiosperms. There are many questions to be answered about the aperture pathway. Characterizing the function of known genes in species from relevant angiosperm groups will allow a better assessment of the functional conservation of these genes in the phylogenetic scale of flowering plants. Of particular importance would be the identification of new factors involved in aperture formation. Characterization of proteins interacting with INP1 and/or D6PKL3, as well as genes responsible for abnormal aperture systems, such as the recently discovered *macaron* and *doughnut* mutants (Plourde et al., 2019), or the regulatory genetic network, would help to better understand the process of aperture formation and its evolution in angiosperms.

## Data Availability Statement

The original contributions presented in the study are publicly available. These data can be found here: EBI repository, accession numbers: ERS6376182, ERS6376183, ERS6376184, ERS6376185, ERS6376186, and ERS6376187.

## Author Contributions

IM-A performed the experiments and analyzed the transcriptomic data with the help of BK and DH. IM-A and VS-S analyzed the functional study data. AD provided critical information and confocal images of pollen. VS-S and AR-G conceived and designed the studies. VS-S, IM-A, and AD drafted the manuscript and all the authors participated in the editing of the manuscript.

## Conflict of Interest

The authors declare that the research was conducted in the absence of any commercial or financial relationships that could be construed as a potential conflict of interest.

## Footnotes

1 https://phytozome.jgi.doe.gov/pz/portal.html

2 https://bioinformatics.tugraz.at/phytometasyn/

3 http://www.bioinformatics.babraham.ac.uk/projects/fastqc/

4 https://github.com/FelixKrueger/TrimGalore

5 ftp://ftp.kazusa.or.jp/pub/eschscholzia/ECA_r1.0.cds.fa.gz

6 https://ccb.jhu.edu/software/hisat2/

7 https://ccb.jhu.edu/software/stringtie/

8 https://www.ncbi.nlm.nih.gov/assembly/GCF_003573695.1

9 https://data.broadinstitute.org/Trinity/Trinotate_v3_RESOURCES/uniprot_sprot.pep.gz
