# Supplementary material for: The Role of INAPERTURATE POLLEN1 as a Pollen Aperture Factor Is Conserved in the Basal Eudicot Eschscholzia californica (Papaveraceae)
Source: Front Plant Sci. 2021 Jul 7;12:701286. doi: 10.3389/fpls.2021.701286 (PMC8294094; doi:10.3389/fpls.2021.701286)
Supplement: Supplementary file 1 [file Data_Sheet_1.PDF]

Supplementary Material

Supplementary Figure 1

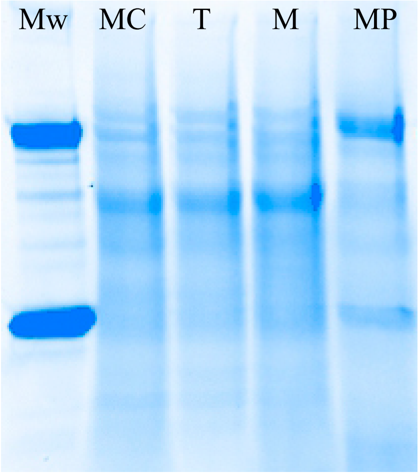

Supplementary Figure 1. Sodium dodecyl sulphate–polyacrylamide gel electrophoresis (SDS-PAGE) of *Eschscholzia californica* proteins visualized by Stain-free technology using a Gel DocTM EZ System. MC, mother cell; T, tetrad; M, microspore; MP, mature pollen.

Supplementary Figure 2

```
                                   N-terminal                  DOG1
                                   ....|....|....|....|....|....|....|....|....|....|
Eca_sc100701.1_g3140.1_EGD_DNA     MIKAAARFGRKKSTRLFKDFYLEWIETLKTNLLPLLRRSILVSSSNQLST
Eca1_DNA                           .....................V............................
Eca2_DNA                           .....................V............................
Eca3_DNA                           .....................V............................
Eca4_DNA                           ..................................................
Eca5_DNA                           .....................V............................
Eca6_DNA                           ..................................................
Eca7_DNA                           ..................................................
Eca9_DNA                           ..................................................
Eca10_DNA                          ..................................................
Eca25_DNA                          ??????????????????...V............................
LT840341_ENA_cDNA                  .....................V............................
ERXG2062521_1KP_cDNA               ..................................................
Eca_TRINITY_DN96950_c3g1i1_cDNA    ..................................................
Eca_TRINITY_DN96950_c3g1i2_cDNA    ..................................................
Eca_rho6_cDNA                      ..................................................
Eca_rho10_cDNA                     ..................................................
Eca_foe6_cDNA                      ..............................D...................
Eca_foe8_cDNA                      ..................................................
Eca_foe9_cDNA                      ..................................................
Eca_foe10_cDNA                     ......................G...........................
Eca2_cDNA                          ..................................................
Eca3_cDNA                          ..............................D...................
Eca4_cDNA                          ..................................................
Eca9_cDNA                          ..................................................
Eca_TRINITY_DN96950_c3g1i5_cDNA    ..................................................
Eca_TRINITY_DN96950_c3g1i8_cDNA    ..................................................
Eca_lut6_cDNA                      ?????????????????????????.........................
Eca_imb37_cDNA                     ???????????????.......G.....R.....................

                                   DOG1
                                   ....|....|....|....|....|....|....|....|....|....|
Eca_sc100701.1_g3140.1_EGD_DNA     HVQMIQHHFQNYYLTLDLAASEDVSQLLFPIWRNSLEKPFLWVGDFHPNL
Eca1_DNA                           ..................................................
Eca2_DNA                           ..................................................
Eca3_DNA                           ..................................................
Eca4_DNA                           ..................................................
Eca5_DNA                           ..................................P...............
Eca6_DNA                           ..................................P...............
Eca7_DNA                           ..................................P...............
Eca9_DNA                           ..................................P...............
Eca10_DNA                          ..................................P...............
Eca25_DNA                          ..................................................
LT840341_ENA_cDNA                  ..................................................
ERXG2062521_1KP_cDNA               ..........................I.......................
Eca_TRINITY_DN96950_c3g1i1_cDNA    ..........................I.......................
Eca_TRINITY_DN96950_c3g1i2_cDNA    ..........................I.......................
Eca_rho6_cDNA                      ....................................G.............
Eca_rho10_cDNA                     ....................................G.............
Eca_foe6_cDNA                      ..................................................
Eca_foe8_cDNA                      ..........................I.......................
Eca_foe9_cDNA                      ..........................I.......................
Eca_foe10_cDNA                     ..........................I.......................
Eca2_cDNA                          ..........................I.......................
Eca3_cDNA                          ..................................................
Eca4_cDNA                          ..........................I.......................
Eca9_cDNA                          ....................................G.............
Eca_TRINITY_DN96950_c3g1i5_cDNA    ..........................I.......................
Eca_TRINITY_DN96950_c3g1i8_cDNA    ..........................I.......................
Eca_lut6_cDNA                      ....................................G.............
Eca_imb37_cDNA                     ..........................I.....C.................
```

```
                                  DOG1 | Acidic | Middle
                                  ....|....|....|....|....|....|....|....|....|....|
Eca_sc100701.1_g3140.1_EGD_DNA    FTNLLRSFLNNN-SSDEEINDTHIEKSSNFPLVWKYPSKNLMNKIEQIEC
Eca1_DNA                          ............-......DN.............................
Eca2_DNA                          ............-......D..............................
Eca3_DNA                          ............-......D..............................
Eca4_DNA                          ............-......D..............................
Eca5_DNA                          ............-......DN.............................
Eca6_DNA                          ............-......D..............................
Eca7_DNA                          ............-......DN.............................
Eca9_DNA                          ............-......DN.............................
Eca10_DNA                         ............-......DN.............................
Eca25_DNA                         ............-.....................................
LT840341_ENA_cDNA                 ............-......DN.............................
ERXG2062521_1KP_cDNA              ............N......D..............................
Eca_TRINITY_DN96950_c3g1i1_cDNA   ............N......D..............................
Eca_TRINITY_DN96950_c3g1i2_cDNA   ............N......D..............................
Eca_rho6_cDNA                     ............-.....................................
Eca_rho10_cDNA                    ............-.....................................
Eca_foe6_cDNA                     ............-.....................................
Eca_foe8_cDNA                     ............N......D.................E...........
Eca_foe9_cDNA                     ............N......D.................E...........
Eca_foe10_cDNA                    ............N......D.................E...........
Eca2_cDNA                         ............N......D..............................
Eca3_cDNA                         ............-.....................................
Eca4_cDNA                         ............N......D.................E...........
Eca9_cDNA                         ............-.....................................
Eca_TRINITY_DN96950_c3g1i5_cDNA   ............N......D..............................
Eca_TRINITY_DN96950_c3g1i8_cDNA   ............N......D..............................
Eca_lut6_cDNA                     ............-.....................................
Eca_imb37_cDNA                    ............N......D.................E...........
```

```
                                  Middle | C-terminal
                                  ....|....|....|....|....|....|....|....|....|....|
Eca_sc100701.1_g3140.1_EGD_DNA    GLRSMVPTLVTRYRKSQSKFLDKCGLNWINCESKQEILKTVEKDLMVEIE
Eca1_DNA                          ................H.................................
Eca2_DNA                          ..................................................
Eca3_DNA                          ..................................................
Eca4_DNA                          ..................................................
Eca5_DNA                          ................H.................................
Eca6_DNA                          ..................................................
Eca7_DNA                          ................H.................................
Eca9_DNA                          ................H.................................
Eca10_DNA                         ................H.................................
Eca25_DNA                         ...............A................................T.
LT840341_ENA_cDNA                 ................H.................................
ERXG2062521_1KP_cDNA              ..................................................
Eca_TRINITY_DN96950_c3g1i1_cDNA   ..................................................
Eca_TRINITY_DN96950_c3g1i2_cDNA   ..................................................
Eca_rho6_cDNA                     ..................................................
Eca_rho10_cDNA                    ..................................................
Eca_foe6_cDNA                     ..................................................
Eca_foe8_cDNA                     ..................................................
Eca_foe9_cDNA                     ..................................................
Eca_foe10_cDNA                    ..................................................
Eca2_cDNA                         ..................................................
Eca3_cDNA                         ..................................................
Eca4_cDNA                         ..................................................
Eca9_cDNA                         ..................................................
Eca_TRINITY_DN96950_c3g1i5_cDNA   ..................................................
Eca_TRINITY_DN96950_c3g1i8_cDNA   ..................................................
Eca_lut6_cDNA                     ..................................................
Eca_imb37_cDNA                    ..................................................
```

```
                                       C-terminal
                                   ....|....|....|....|....|....|....|....|....|....|
Eca_sc100701.1_g3140.1_EGD_DNA     ELVGVFLDANRLRRSVLTEIIGATDIYQAALYLEGLAQFFVGFSDGELLH
Eca1_DNA                           ..................................................
Eca2_DNA                           ..................................................
Eca3_DNA                           ..................................................
Eca4_DNA                           ..................................................
Eca5_DNA                           ..................................................
Eca6_DNA                           ..................................................
Eca7_DNA                           ..................................................
Eca9_DNA                           ..................................................
Eca10_DNA                          ..................................................
Eca25_DNA                          ..................................R...............
LT840341_ENA_cDNA                  ..................................................
ERXG2062521_1KP_cDNA               ..................................................
Eca_TRINITY_DN96950_c3g1i1_cDNA    ..................................................
Eca_TRINITY_DN96950_c3g1i2_cDNA    ..................................................
Eca_rho6_cDNA                      ..................................................
Eca_rho10_cDNA                     ..................................................
Eca_foe6_cDNA                      ..................................................
Eca_foe8_cDNA                      ..................................................
Eca_foe9_cDNA                      ..................................................
Eca_foe10_cDNA                     ..................................................
Eca2_cDNA                          ..................................................
Eca3_cDNA                          ..................................................
Eca4_cDNA                          ..................................................
Eca9_cDNA                          ..................................................
Eca_TRINITY_DN96950_c3g1i5_cDNA    ..................................................
Eca_TRINITY_DN96950_c3g1i8_cDNA    ..................................................
Eca_lut6_cDNA                      ..................................................
Eca_imb37_cDNA                     ..................................................

                                   C-terminal
                                   ....|....|....|
Eca_sc100701.1_g3140.1_EGD_DNA     EFEQCKIPLSVE---
Eca1_DNA                           ..........GI---
Eca2_DNA                           ..........GI---
Eca3_DNA                           ..........GI---
Eca4_DNA                           ..........GI---
Eca5_DNA                           ..........GI---
Eca6_DNA                           ..........GI---
Eca7_DNA                           ..........GI---
Eca9_DNA                           ..........GI---
Eca10_DNA                          ..........GI---
Eca25_DNA                          ......?????????
LT840341_ENA_cDNA                  ..........GI---
ERXG2062521_1KP_cDNA               ..........?????
Eca_TRINITY_DN96950_c3g1i1_cDNA    ............---
Eca_TRINITY_DN96950_c3g1i2_cDNA    ............---
Eca_rho6_cDNA                      ..........GI---
Eca_rho10_cDNA                     ..........GI---
Eca_foe6_cDNA                      ..........GI---
Eca_foe8_cDNA                      ..........GI---
Eca_foe9_cDNA                      ..........GI---
Eca_foe10_cDNA                     ..........GI---
Eca2_cDNA                          ..........GI---
Eca3_cDNA                          ..........GI---
Eca4_cDNA                          ..........GI---
Eca9_cDNA                          ..........GI---
Eca_TRINITY_DN96950_c3g1i5_cDNA    ..........GI---
Eca_TRINITY_DN96950_c3g1i8_cDNA    ..........GHLGS
Eca_lut6_cDNA                      ???????????????
Eca_imb37_cDNA                     ???????????????
```

Supplementary Figure 2. Protein alignment of 29 sequences of *Eschscholzia californica* INP1 (EcINP1). The alignment comprises 265 positions. The subdivisions of EcINP1 are shown according to Li *et al*. (2018). The possition of the introns is marked with a red triangle at the top of the alignment. 26 sequences were generated by us and three were downloaded from databases (indicated after the locus name: EGD, *Eschscholzia* Genome Database; ENA, European Nucleotide Archive; 1KP, 1000 Plants Project). 11 sequences come from the translation of gDNA and 18 from cDNA (indicated at the end of the name : DNA *vs* cDNA). As a reference sequence we have taken the sequence of the scaffold Eca_sc100701.1_g3140.1 obtained from the *Eschscholzia* Genome database. The named sequences starting with Eca_TRINITY come from transcriptomes obtained by us.

Supplementary Figure 3

A

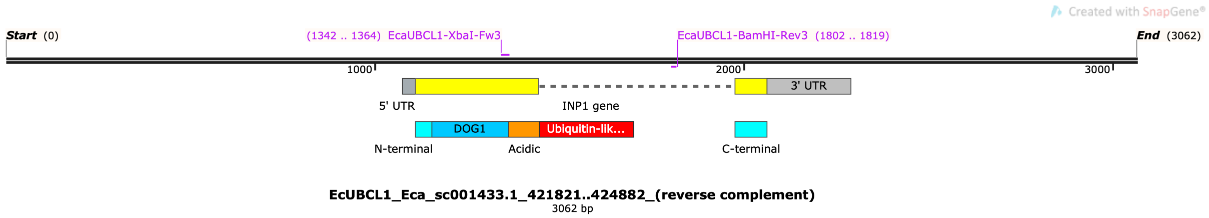

B

***EcUBCL1*_Eca_sc001433**
***EcINP1*_Eca_sc100701**

```
                10        20        30        40        50        60        70        80        90       100
        ....|....|....|....|....|....|....|....|....|....|....|....|....|....|....|....|....|....|....|....|
EcUBCL1 CAGCAAGTAGCAAAATTATAAATTCAGCTGACGGTTGCTTCTTTTAGCAAGAGTCTCAATCAGTCTCACTTAGCACACCTTAACCAGGTACTTTTATCTG
EcINP1  ----------------------------------------------------------------------------------------------------

               110       120       130       140       150       160       170       180       190       200
        ....|....|....|....|....|....|....|....|....|....|....|....|....|....|....|....|....|....|....|....|
EcUBCL1 CTTTCTAAAAATAACTAATAAATTTCAATAACGTAGCGTCGACGATATTGATTGTGCCATCAATGCTTCGAGGGGGACTTTGGGCAAAGACTTAGTGAAT
EcINP1  ----------------------------------------------------------------------------------------------------

               210       220       230       240       250       260       270       280       290       300
        ....|....|....|....|....|....|....|....|....|....|....|....|....|....|....|....|....|....|....|....|
EcUBCL1 GATTGTACCAATTCCTAAGAGAATAAGTTGTGTTTTAACTATCCAATGTTAATAAATGGCTTTATGAGCGAGGTGTAATTTGTTGTATGAAAAATATTGC
EcINP1  -----------------------------------------------------TCGGCTTTGATGAATTTTGAATTGTGATCAAGCATAACCAGTCCAAT

               310       320       330       340       350       360       370       380       390       400
        ....|....|....|....|....|....|....|....|....|....|....|....|....|....|....|....|....|....|....|....|
EcUBCL1 GAATCAAATTGTCGAAATTTATTGTTTGAGTTTGGTTTAATCTTCATAAGATTCATTTAGACCTCCAAATATGGTGTTAACATTTGATTAGTTAGACTGC
EcINP1  TCCTATCTTCTGTTAAGAGAAAGAGAAAGATGCCTCGACATTAATCTTATAGCGGAGCGGACGTGGGGGTCCGTCTTTCCCGCTTTCAAGTATTACTGTA

               410       420       430       440       450       460       470       480       490       500
        ....|....|....|....|....|....|....|....|....|....|....|....|....|....|....|....|....|....|....|....|
EcUBCL1 ACCCACATCTTCTAGTTATCATATCATATAGCATAGATAGAGATCAAAAGATGAAATGAGCACATAACAACTACAAAACGTAGTATGGCCTTGACACGTC
EcINP1  GCTAATCTTGAGAGATTTAGTCGTCTTGCGCTTCATGTGTAAAATTAAAAGAGTTAATTGTCTTTATATTGTCCTTCGAATTTAGCTGGTTATGTTGCAG

               510       520       530       540       550       560       570       580       590       600
        ....|....|....|....|....|....|....|....|....|....|....|....|....|....|....|....|....|....|....|....|
EcUBCL1 AAGAGCATGAAATAAGCTCATAAACCATCATGAAAACAAAGACCTAGAAACGTATCCTGATGATCATGACACTAAATTCTACCTGCCATCAGGAACTAGT
EcINP1  ATAGATCCGAAGACGAGGCTTTCTACATATACCAAACACTCATGGCCAACAAGAAGAGATTAATAGCAGCGTTATCGTTATCTAAGTGTTTCCACGAGCA

               610       620       630       640       650       660       670       680       690       700
        ....|....|....|....|....|....|....|....|....|....|....|....|....|....|....|....|....|....|....|....|
EcUBCL1 TTGTTTTTTAATATACATATTTAGAAGGTTAACTATCAACTCTACGACTGCAGTCCATTACCACCCCCGATGGTAGTTAATAGATTATGAAACTGGAGTG
EcINP1  TATCCATTGGGTGAGGTCGTCATTTGATCTGAAAATATTTCAATAAAACGTTAAACTTCTAGCAAATTGGACTTCTTATCTCACGTTCATTATCTCACAT

               710       720       730       740       750       760       770       780       790       800
        ....|....|....|....|....|....|....|....|....|....|....|....|....|....|....|....|....|....|....|....|
EcUBCL1 ATTATTAGAGTCTTCTGCACCTTAATTTATAATATTGAGGTGACAGAAAGTAGAAACATATCAAGGAGATTAAGGGTCTGTTTGATACGTGAAAAAAGTG
EcINP1  TATCAAAGCTTCGTTGATGTTAAGACACTAGCGATCTTCTCCTTCTTCGTGACAGACCAGATCTAGTATCAAAAAAAAAAAACATCGATATCATCATCGC
```

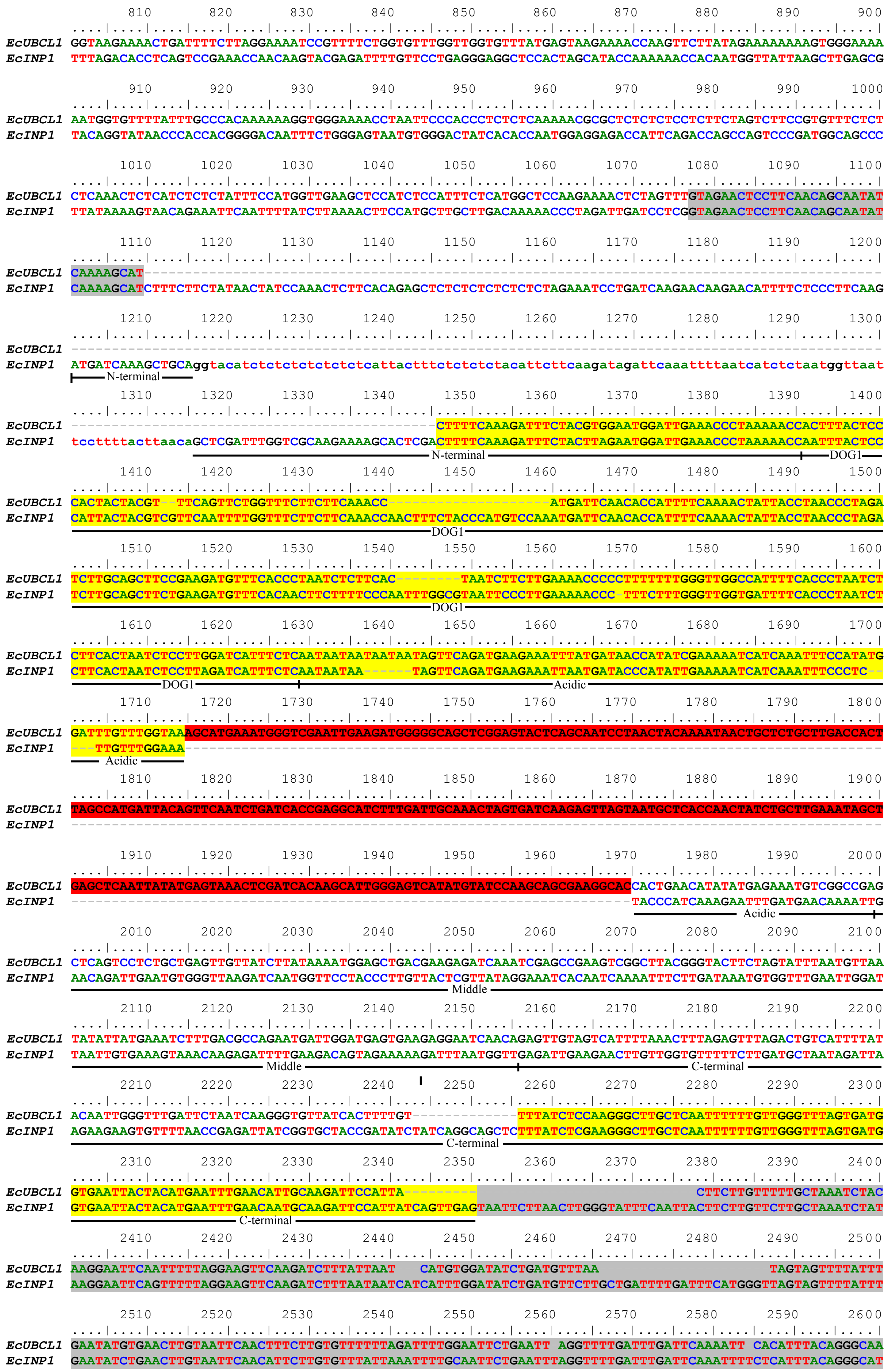
EcUBCL1
EcINP1
N-terminal
DOG1
Acidic
Middle
C-terminal

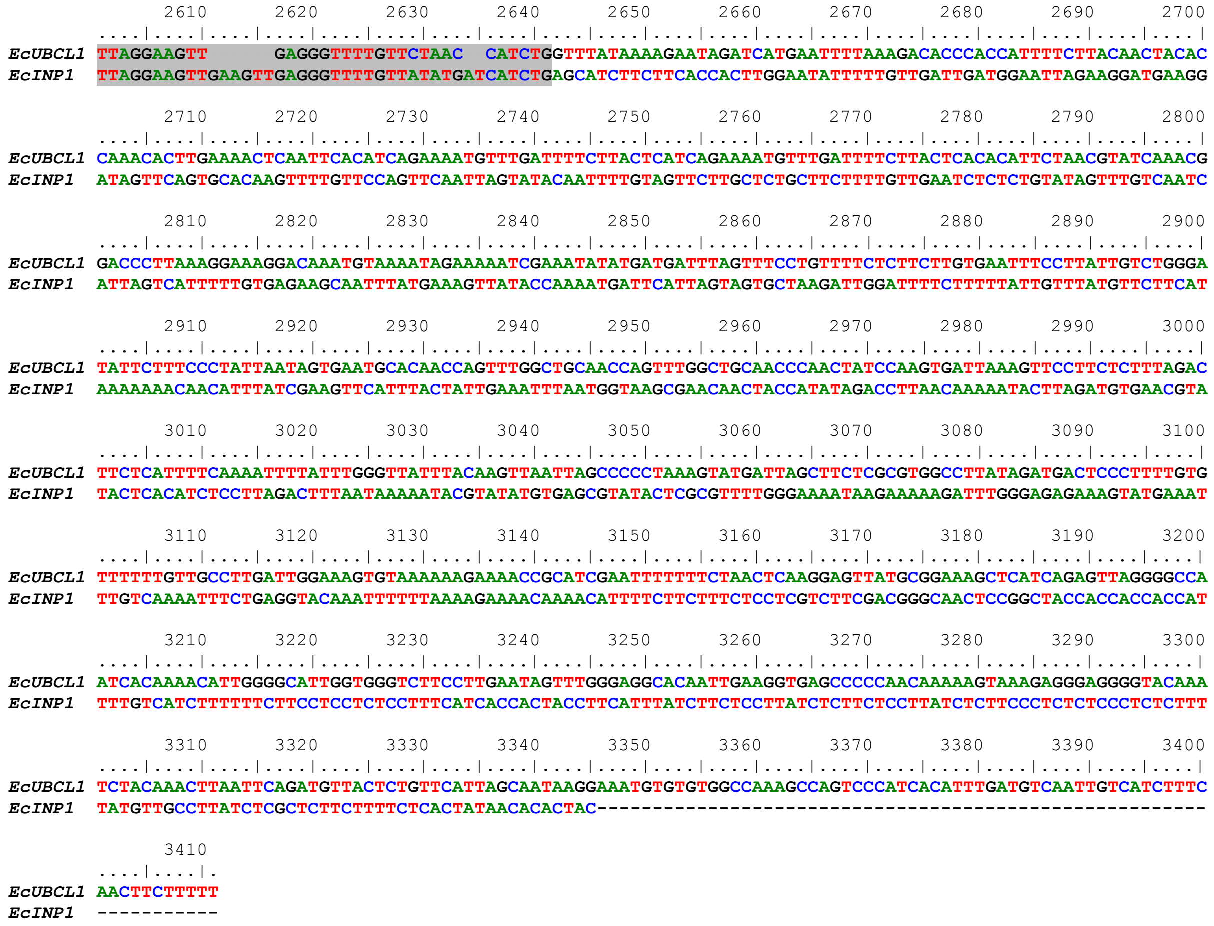

Supplementary Figure 3. The *EcUBCL1* sequence from *Eschscholzia californica* containing a fragment from the ubiquitin-like domain-containing CTD phosphatase and two dispersed fragments of *EcINP1*. The sequence shown corresponds to the reverse complement of the scaffold Eca_sc001433.1 (positions 421821..424882) obtained from the *Eschscholzia* Genome database. **(A)** Sequence map, created with SnapGene Viewer 5.2.1, showing the fragments with identity to the *EcINP1* gene (coding sequences, yellow; UTR's, grey) and the ubiquitin-like domain-containing CTD phosphatase region (red). Below the *EcINP1* coding regions, the domains that comprise them are specified. Primers used for VIGS experiment are shown above the sequence. **(B)** Alignment of *EcUBCL1* and *EcINP1* (reverse complement of the scaffold Eca_sc100701.1 positions 1579981..1582805 from the *Eschscholzia* Genome database). The shaded fragments in both sequences correspond to the regions that share significant identity (UTRs, grey; coding regions, yellow). Red shading in *EcUBCL1* indicates the sequence from a ubiquitin-like domain-containing CTD phosphatase. The *EcINP1* CDS is underlined and its domains are indicated; the sequence of the intron is indicated in lower case letters.

Supplementary Figure 4

A

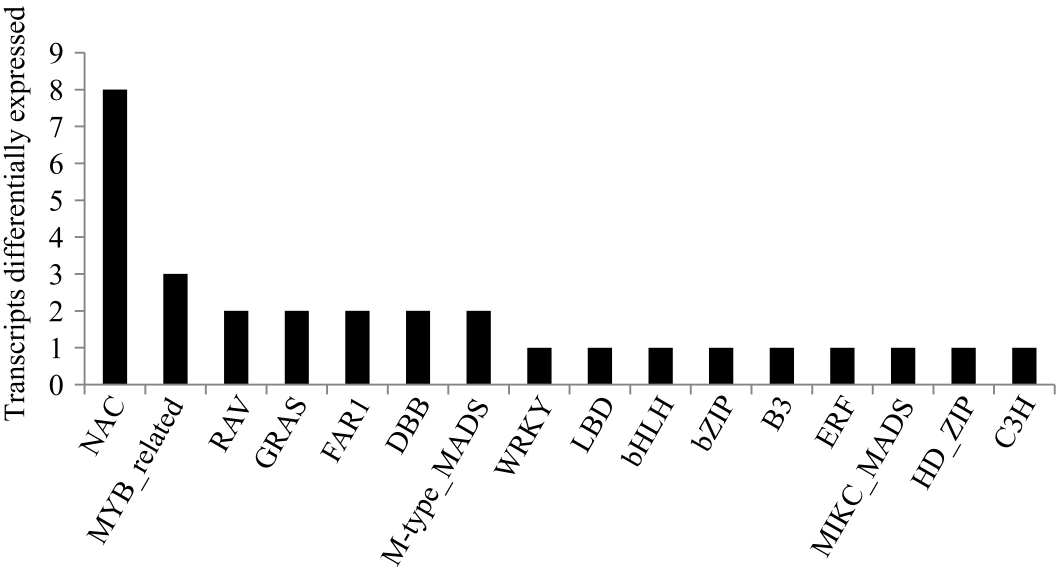

B

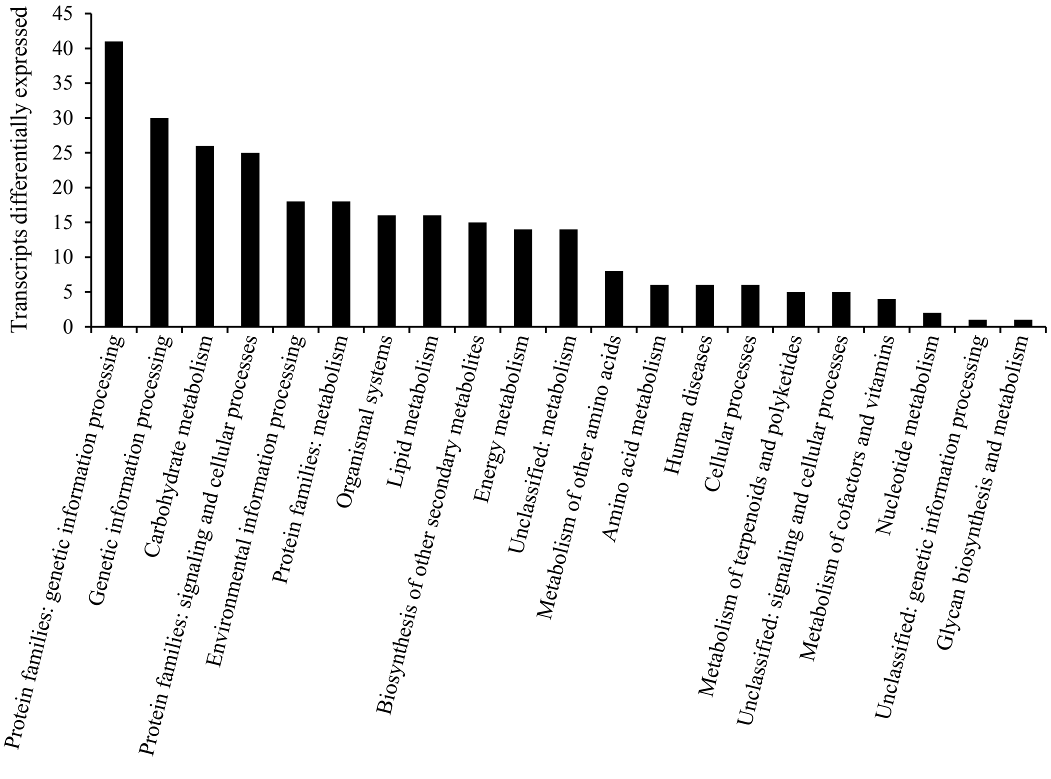

Supplementary Figure 4. Functional annotation of DEGs from VIGS-silenced plants of *Eschscholzia californica*. **(A)** Histogram showing the distribution by transcription factor families of the 30 transcription factors identified from Plant Transcription Factor DataBase. **(B)** Classification of each DEG into KEGG functional categories, using GhostKoala mapping tool.

Supplementary Figure 5

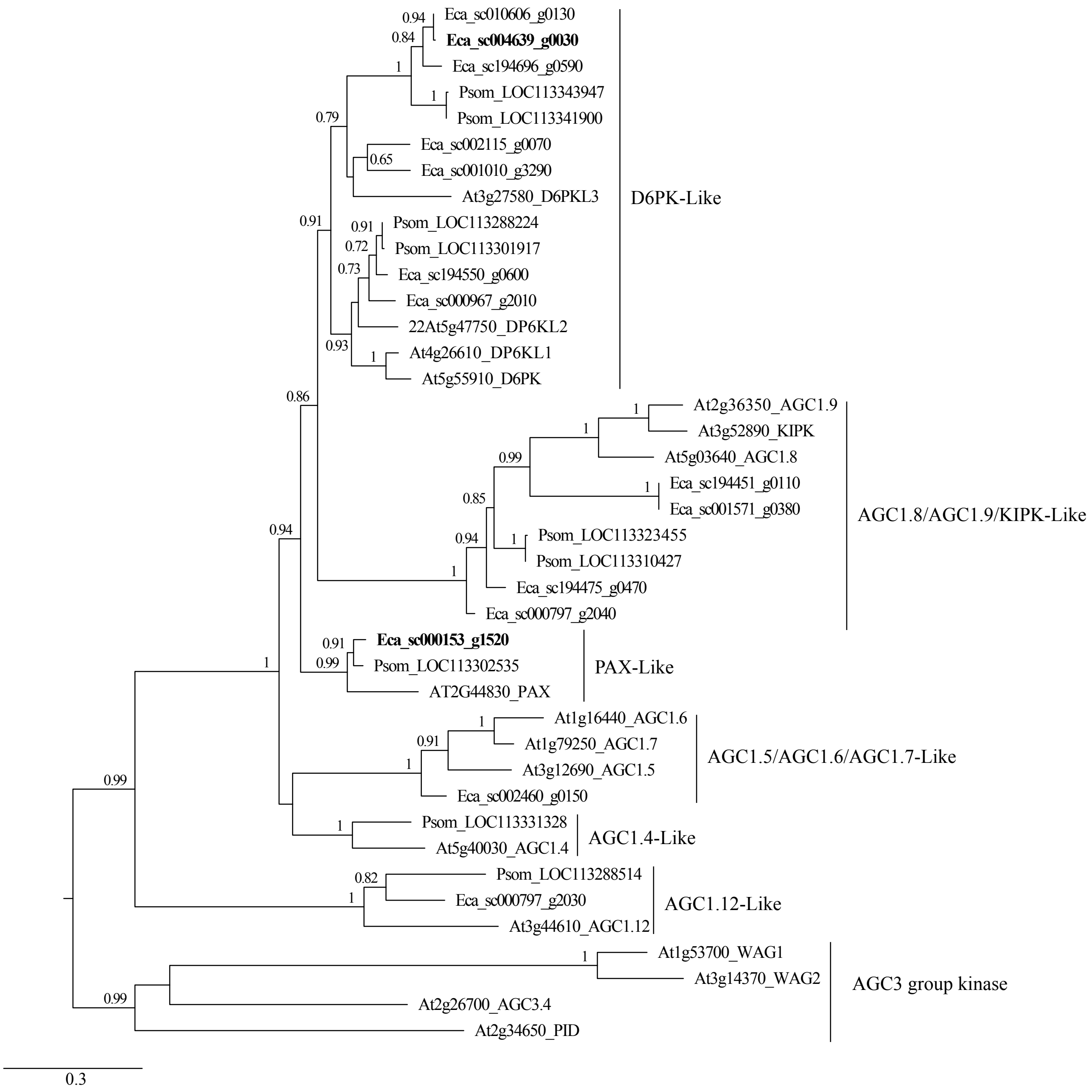

Supplementary Figure 5. Maximum likelihood tree for the AGC1 group kinases, based on the protein sequences of the kinase domain, from *Eschscholzia californica* (Eca), *Papaver somniferum* (Psom), and *Arabidopsis thaliana*. The locus identifier is shown for each sequence. The sequences of the DEGs found in *E. californica* are in bold type. The sequences of the AGC3-group kinases from *A. thaliana* were used as an outgroup. The types of kinases indicated on the right for each clade are listed according to the names of the *A. thaliana* kinases.

Supplementary Figure 6

A

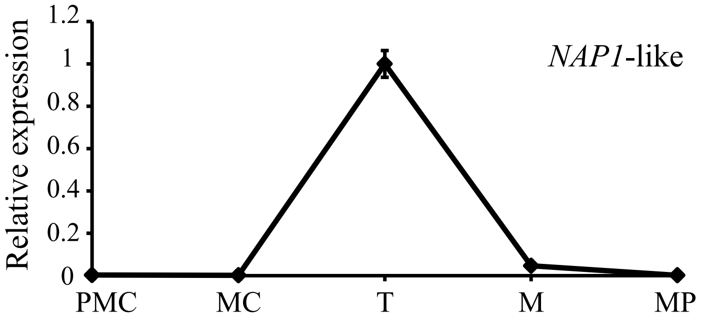

B

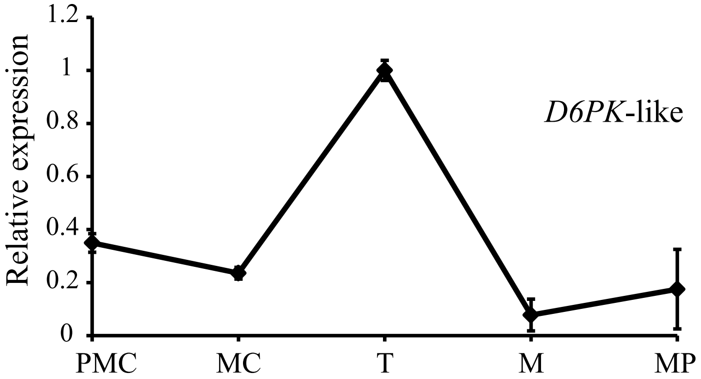

C

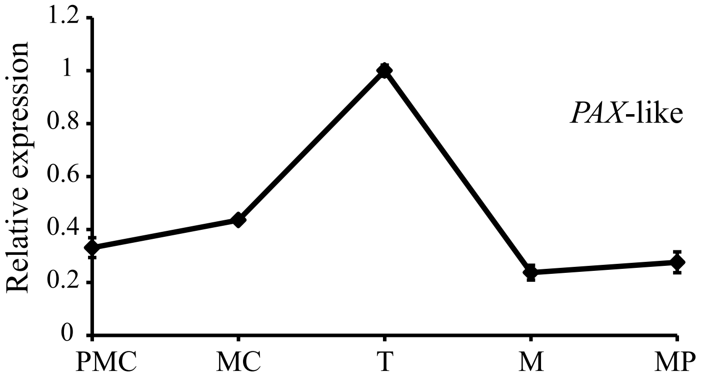

Supplementary Figure 6. qRT-PCR-based expression analysis of **(A)** *NAP1*-like, Eca_sc004324.1_g2710.1; (**B**) *D6PK*-like, Eca_sc004639.1_g0030.1; **(C)** *PAX*-like, Eca_sc000153.1_g1520.1. The expression patterns were tested in anthers at different pollen development stages. Actin was used as a normalization control. PMC, pre-mother cell; MC, mother cell; T, tetrad; M, microspore; MP, mature pollen.

Supplementary Table 1. Primer sequences used in this study.

Primer	Sequence	Purpose
EcaINP1-F	ATGATCAAAGCTGCAGCTCGA	Intraspecific variability and phylogeny
EcaINP1-R	AATGCCTGATAATGGAATCTTGC	Intraspecific variability and phylogeny
EcaINP1-F2-EcoRI	GGAA_GAAT_TCCCTTCAAGATGATCAAAGCTGC	VIGS construct
EcaINP1-R2-BamHI	GGAA_GGATCC_GGAACCATTGATCTTAACCCACA	VIGS construct
EcaINP1-FwReal2	GCTGCAGCTCGATTTGGT	Gene expression qRT-PCR and semi-qRT-PCR
EcaINP1-RvReal4	AGAAACCAGAATTGAACGACGT	Gene expression qRT-PCR
EcaINP1-RvReal3	TGGACATGGGTAGAAAGTTGG	Gene expression semi-qRT-PCR
Actin2RTQFw	TTACAATGAGCTTCGTGTTGC	Gene expression qRT-PCR
Actin2RTQRv	CCCAGCACAATACCTGTAGTAC	Gene expression qRT-PCR
WAK2qPCRFw2	CCTTTCGTGGGATAACCGCT	Gene expression qRT-PCR
WAK2qPCRRv2	ACTCGACTTGACGTCCCTATG	Gene expression qRT-PCR
PAX2qPCRFw2	AGCGGCACTAGGTGCTATTT	Gene expression qRT-PCR
PAX2qPCRRv2	CTCTTTCTGTCTGAGCTCTGGT	Gene expression qRT-PCR
WAK3qPCR2Fw1	TCGAATGGGACCTTGCACC	Gene expression qRT-PCR
WAK3qPCRRv1	TCCTGCGGTTTCTGTAGCAAT	Gene expression qRT-PCR
NAC25qPCRFw2	GGACATGACGCACACTGCTT	Gene expression qRT-PCR
NAC25qPCRRv2	ACATCATCAGCGGGTTCTTG	Gene expression qRT-PCR
D6PKL1qPCRFw2	CTCATGTTTCATGCCGCGAT	Gene expression qRT-PCR
D6PKL1qPCRRv2	GCAATTAACTCAGGGCGTGC	Gene expression qRT-PCR
PAX_qPCR_Fw2	TCGAAGGTGTGAATTGGGCA	Gene expression qRT-PCR
PAX_qPCR_Rv2	CCCCAACCGGATCAACTGAA	Gene expression qRT-PCR
NAP1_qPCR_Fw2	TATGGGGTGGACCATACGGA	Gene expression qRT-PCR
NAP1_qPCR_Rv2	TTGTTGGAATGGGAACCAGTCT	Gene expression qRT-PCR
ANXUR1_qPCR_Fw2	TGTGATCACGGAGTTCAGCG	Gene expression qRT-PCR
ANXUR1_qPCR_Rv2	GTTCTTCACCTCCGGGGTTT	Gene expression qRT-PCR
Rho_qPCR_Fw2	GAAGAGTCGGTGGTCGCTTT	Gene expression qRT-PCR
Rho_qPCR_Rv2	AGTGTACCAGTAGAAGCAGCG	Gene expression qRT-PCR
NADPH_qPCR_Fw	ACTGCTCCACTCAAGAACGC	Gene expression qRT-PCR
NADPH_qPCR_Rv	ACCACCAGCGCTTACGATAG	Gene expression qRT-PCR
EcaUBCL1-XbaI-Fw3	GGAA_TCTAGA_AATCTCCTTGGATCATTTCTCAA	VIGS construct
EcaUBCL1-BamHI-Rv3	GGAA_GGATCC_AGAAGTACCCGTAAGCCG	VIGS construct

Supplementary Table 2. List of sequences used to test homology of the *Eschscholzia californica* EcINP1 protein by constructing the gene tree shown in Figure 1.

**Species**	**Locus name**	**Database**	**NCBI-ENA accession number (transcript/protein)**
Aquilegia coerulea	Aqcoe7G079000.1	Phytozome	
	Aqcoe7G079000.2	Phytozome	
Anemone pulsatilla	UPOG-2056161	1KP	
Arabidopsis thaliana	INP1 // AT4G22600	NCBI	NM_118386<br>NP_193991
Argemone mexicana	IRAF-2015101	1KP	
Brachipodium distachium	LOC100826863 // Bradi3g50820	NCBI	XM_024460675<br>XP_024316443
Eschscholzia californica	Eca_sc100701.1_g3140.1	Eschscholzia Genome Database	
	Eca TRINITY DN96950 c3 g1 i5	ENA	OU070349
Macleaya cordata	BVC80_1727g16	NCBI	MVGT01000886<br>OVA15152
Nandina domestica	YHFG-2062185/2005708/2005709	1KP	
Nelumbo nucifera	LOC104598573	NCBI	XM_010260712<br>XP_010259014
Nicotiana tomentosiformis	LOC104092836	NCBI	XM_009598514.3<br>XP_009596809
Oryza sativa	LOC4330222	NCBI	XM_026022761<br>XP_025878546
Papaver rhoeas	BEKN-2065739_GMAM-2014596	1KP	
Papaver somniferum	LOC113359130	NCBI	XM_026602809<br>XP_026458594
Papaver somniferum	LOC113284312	NCBI	XM_026533749<br>XP_026389534
Populus trichocarpa	LOC18093990 // POPTR_001G120700v3	NCBI	XM_024587580<br>XP_024443348
Solanum lycopersicum	LOC101247949	NCBI	XM_004245692.4<br>XP_004245740
Thalictrum thalictroides	KAF5188586	NCBI	JABWDY010026581<br>KAF5188586
Theobroma cacao	LOC18606310	NCBI	XM_007039852.2<br>XP_007039914
Vancouveria hexandra	vhe_VHALF1JP_Trinity_comp81110 c0_seq1	Phytometasyn	
Vitis vinifera	LOC100250621	NCBI	XM_010648242.2<br>XP_010646544
Zea mays	LOC100191973 // GRMZM2G112914	NCBI	XM_020553019<br>XP_020408608

Supplementary Table 3. Summary of sequencing and assembly for *EcINP1*-silenced and wild-type *Eschscholzia californica*.

	Wild 1	Wild 2	Wild 3	VIGS 1	VIGS 2	VIGS 3	Average
Sequenced reads	40072074	43677076	43973604	67984350	57866512	50223034	50632775
GC content	42	42.76	41.9	42.62	42.27	41.9	42.24
Q20(%)	98.13	97.87	98.04	97.83	97.68	97.74	97.88
Q30(%)	94.39	94.03	94.32	93.69	93.32	93.45	93.87
No HQ reads	24.482	61.764	50.048	113.308	83.572	62.008	65.864
HQ reads	40047592	43615312	43923556	67871042	57782940	50161026	50566911
Paired reads	20023796 (100%)	21807656 (100%)	21961778 (100%)	33935521 (100%)	28891470 (100%)	25080513 (100%)	25283456
Aligned paired reads	10344293 (51.66%)	11185146 (51.29%)	11740766 (53.46%)	18698472 (55.1%)	15913421 (55.08%)	13698976 (54.62%)	13596846 (53.53%)
Number of contigs >200 bases (merged assemble)	34729						
Average length of contigs	1006						
